# A systematic approach to determine the outcome of the competition between two microbial species in bioreactor cocultures

**DOI:** 10.1007/s10482-024-02035-y

**Published:** 2024-11-14

**Authors:** Marcin Bizukojć, Tomasz Boruta, Anna Ścigaczewska

**Affiliations:** Department of Bioprocess Engineering, Faculty of Process and Environmental Engineering, Lodz University of Technology, ul. Wólczańska 213, 93-005 Lodz, Poland

**Keywords:** Fungi, Actinomycetes, Bioreactor, Coculture, Dominance

## Abstract

The two-species microbial cocultures are effective in terms of awakening the cryptic biosynthetic pathways. They may also lead to the improved production of previously discovered molecules. Importantly, only a few outcomes of the cocultures may prove desirable, namely those leading to the formation of useful secondary metabolites. To address this issue, a method allowing for the evaluation of the final outcome of the co-culture process and fine-tune the cocultivation strategy was proposed. The systematic approach was supported by the experimental data from the bioreactor runs with the participation of *Aspergillus terreus* and *Penicillium rubens* confronted with *Streptomyces rimosus* and *Streptomyces noursei*. Kinetic, morphological and metabolic aspects of dominance were analysed via the newly proposed formula describing the dominance pattern. The suggested method involved the determination of the numerical value representing the dominance level. When it was high (value 1) no useful metabolites were formed apart from those originating from the winning counterpart. But either for the partial dominances or when the winning organism changed within the run or when the competition ended in draw, the number of the secondary metabolites of interest in the broth was the highest. Next, the systematic approach illustrated how the delayed inoculation strategy influenced the level of dominance leading to the change of winning counterpart and the set of metabolites produced. The proposed systematic approach allows for the reliable determination of the level of dominance in the two-species cocultures to seek for the potentially useful substances for future applications.

**Supplementary Information:**

The online version contains supplementary material available at 10.1007/s10482-024-02035-y.

## Introduction

Secondary metabolites are an important group of biotechnological products. Their relevance is of pharmaceutical nature, as they display bioactivities demanded in the context of drug discovery (Demain and Fang 2000; Hegemann et al. 2023). Despite years of studies and revealing hundreds of new substances of natural origin, the need for the novel biologically active secondary metabolites remains unabated (Keller 2019; Walesch et al. 2023). The natural, including microbial, sources of secondary metabolites are irreplaceable as it is often a “mission impossible” task to mimic their biosynthesis with the use of chemical technology tools and methods. But uncovering the full biosynthetic potential of the given microorganism is not a straightforward task as the production of secondary metabolites of the given species depends on the environmental conditions and is a kind of response to changing life conditions in the natural environment. These metabolites play the role of the biochemical weapon against other organisms from the various levels of evolutionary development in the never-ending struggle of a living organism to survive in its ecological niche. To make a given microbial strain produce a wider variety of secondary metabolites the bioprocess-based strategy of using unconventional methods of cultivation (Brakhage 2013; Xu et al. 2022) including microbial cocultures can be employed. In the cocultures the conditions for the interspecies interactions occur and, consequently, they may ultimately lead to the awakening of metabolic pathways that are inactive in the monocultures (Bertrand et al. 2014; Pacheco and Segre 2019; Arora et al. 2020; Ravikrishnan et al. 2020; Knowles et al. 2022; Selegato and Castro-Gamboa 2023).

Furthermore, the stimulation or inhibition of secondary metabolite formation under the co-cultivation conditions is often observed. If any of these metabolites is believed to be a desired one for any industrial purpose the cocultivation may become the method of its overproduction. Technically the cultivation of two-species in one bioreactor is financially affordable, if assumed the full contact of the species in the bioreactor, as the increase of the costs is only connected with the preparation of another inoculum (Goers et al. 2014; Rosero-Chasoy et al. 2021; Kapoore et al. 2021).

Despite its high biosynthetic potential and the technical simplicity of the process, there are several challenges of microbial co-cultivations. They are associated with the experimental design and, above all, the prediction of the final outcome of the cocultivation. It is crucial to know to what an extent one species is capable of dominating its counterpart. Total elimination of one of the species does not need not to be the optimum scenario (Boruta et al. 2020). In the available literature there is no systematic approach of describing and predicting the outcome of such cultivations. Furthermore, even if the outcome of the potential cocultivation is somewhat described and predicted, another question arises how to change the undesired scenario and describe the outcome of the new co-cultivation strategy.

All in all, the issues of the bioreactor-based production of secondary metabolites in the microbial cocultures are greatly underexplored. Only a few papers describe bioreactor co-cultivations, in which the secondary metabolites were produced (Luti and Mavituna 2011; Boruta et al. 2021, 2022, 2023a, b; Ścigaczewska et al 2021) and the majority of cocultures were investigated with the use of shake flasks or even Petri dishes, where the cultivation conditions are not the same as in the aerated and mechanically stirred bioreactors.

The aim of the work was to establish a systematic approach to determine the level of the dominance of the individual microorganisms in the two-species cocultures and to test it upon the large set of experimental data coming from the stirred tank bioreactor experiments with the participation of filamentous fungi and actinomycetes displaying rich secondary metabolism. Next, the outcome of the scenario with delayed inoculation to change the level of dominance was described with the use of this approach.

## Materials and methods

The elaboration of the systematic approach was aimed at the determination of the outcome of the competition between two microbial species in the cocultures grown in the stirred tank bioreactors. At the beginning the systematic approach was proposed. Next, it was applied to evaluate the outcome of the two-species coculture experiments with such filamentous microorganisms as fungi and actinomycetes competing with each other. The cases coming from the previously published experiments that contained the data concerning co-culturing of two fungal and two actinomycete species illustrated the proposed systematic approach.

### Systematic approach

The systematic approach to evaluate the dominance of the individual microorganism growing in the coculture was made in the following sequence:

- The kinetic dominance,
- The morphological dominance,
- The metabolic dominance.

All these analyses were next supported by the experimental data coming from both the coculture bioreactor run and respective monoculture bioreactor runs and dealt with:

- The kinetic data, namely dissolved oxygen curves and carbon source concentration curves,
- The microscopic images and morphological quantitative data,
- The metabolic repertoire of the cultivated species.

### Sources of experimental data required to perform the analysis of dominance

Previously published experimental data concerning the two-species bioreactor cocultures served as the cases to illustrate this systematic approach. These were the cocultures of *Aspergillus terreus* ATCC 20542 and *Streptomyces rimosus* ATCC 10970 (Boruta et al. 2021; Ścigaczewska et al 2021), *Aspergillus terreus* ATCC 20542 and *Streptomyces noursei* ATCC 11455 (Boruta et al. 2022), *Penicillium rubens* ATCC 28089 and *Streptomyces rimosus* ATCC 10970 (Boruta et al. 2023a), *Penicillium rubens* ATCC 28089 and *Streptomyces noursei* ATCC 11455 (Boruta et al. 2023b).

## Results

Comparing the growth of two microbial species, the common knowledge on the growth of microorganisms prompts us that the faster growing organism always defeats the slower growing one. It seems to be true in the majority of cases when two microorganisms compete with each other and there are no positive ecological interactions established. It could sometimes end the considerations without drawing more detailed conclusions and, what is worse, without the full knowledge of the outcome of the process. Furthermore, one can never determine the biomass growth rate of the individual microorganism in the coculture as it is impossible to separate them. If one used carbon substrate curves, although easily determined, it would show the substrate uptake by both species. Thus all studies should be started from the analysis of kinetics but later go further to take both morphological development of the species and metabolic repertoire into account.

### Framework of the analysis

The universal framework of analysis is going to be proposed. It can be applied to any microbial species growing in the bioreactor coculture, albeit its potential limitations were described further.

*The level of dominance* of the given microorganism was described with the use of the following framework presented both in the form of algorithmic graph and the *dominance pattern* formula.

Three *aspects of dominance* were under consideration: kinetic, morphological and metabolic ones. Each of them was analysed with regard to the winner of the one out of two competing species. Thus the win of one organism meant the defeat of the other one. In some cases the microbial competition may have ended in draw.

The following *dominance pattern* formula to evaluate the outcome of microbial competition was proposed:

1 $$\left\{ {{\text{K}}\left( x \right)_{P} ,{\text{ M}}\left( {x.y.z} \right)_{P} ,{\text{ Mt}}\left( {x.y.z} \right)_{P} , \, \left[ {{\text{Mt}}\left( {x.t.n} \right)} \right]_{P} } \right\} \to \left( W \right)$$

*The aspects of dominance* under consideration were denounced respectively: K—kinetic dominance, M—morphological dominance and Mt—metabolic dominance.

Next, the entire characteristics of dominance were taken into account, namely: *x—*level of dominance (win or draw). *y*—characteristics of dominance (positive effect in the given aspect). *z*—characteristics of dominance (negative effect in the given aspect). t—transformed metabolites found. n—new metabolites found.

Ultimately, a winner organism participating in the competition was denoted as W.

The indices in the formula were allowed to have the following values: *x* has the value of 1 for the dominance, value 3 for the draw (value 2 was reserved only for the dominance with new metabolites formation) and *y, z, t, n* was equal either to 0 or 1. Value 0 stood for “no effect”, while 1 for “the effect observed”.

Symbol P was reserved for the name or symbol of the microorganism, to which the formula was referred. The detailed description of the aspects of dominance and their characteristics is presented in Table 1 summarising the whole systematic approach to be presented in this work.

**Table 1:** Determination of *the dominance* in the coculture

	Aspect of dominance	Coculture winner		Coculture loser		Outcome
K(1)	Kinetic dominance	Both curve of dissolved oxygen (DO) level within 24 h and carbon source concentration curve are similar for the coculture and respective winner monoculture		Both curve of dissolved oxygen (DO) level within 24 h and carbon source concentration curve in the coculture fall apart from the respective loser monoculture curves		Growth rate of one microbe is higher than that of its counterpart and the coculture grows with the high rate attributed to the faster microorganism
K(3)	No kinetic dominance	Both curve of dissolved oxygen level (DO) within 24 h and carbon source concentration curve fall apart for the coculture and respective monocultures				Hardly can any conclusions be formulated; it seems to be a draw
M(1.0.0)	Morphological dominance	Agglomerates and dispersed hyphae almost identical with regard to size and shape in the winner monoculture and coculture		No agglomerates of the loser in the coculture. Hardly can any dispersed hyphae be found		Morphological development of the loser is practically blocked
M(1.1.0)	Morphological dominance with single-weakened morphology	Agglomerates and dispersed hyphae almost identical with regard to size and shape in the winner monoculture		In the coculture loser agglomerates smaller than those in the monoculture; dispersed hyphae present		Both species morphologically developed with the affected morphology of the loser
M(1.0.1)	Morphological dominance with double-weakened morphology	Agglomerates and dispersed hyphae not identical with regard to size (smaller) and shape in the winner monoculture and coculture		Loser agglomerates and dispersed hyphae present in the coculture but smaller than those in the monoculture; dispersed hyphae present		Both species morphologically developed, the morphology of the winner and loser is affected
M(3)	Morphological draw	Agglomerates and dispersed hyphae of both species present in the coculture. They are similar with regard to size and shape to their counterparts in the monocultures, nevertheless quantification of dispersed hyphae of both species is here the most misleading				Both species morphologically developed, although the size of the agglomerates of both species can be the same or smaller or bigger and different shape like those ones in the respective monocultures
Mt(1.0.0)	Metabolic dominance	All metabolites produced in the coculture are like those in the winner monoculture with regard to number and quantity		No metabolites in the coculture		Metabolism of the loser is completely blocked and that of the winner practically unaffected
Mt(1.1.0)*	Partial metabolic dominance	Some metabolites of the winner produced in higher number or quantity than those found in the respective monoculture		No metabolites in the coculture		Metabolism of the loser is completely blocked and that of the winner affected by the loser
Mt(1.0.1)*		Some metabolites of the winner produced but in lower number or quantity or even not present compared to those found in the respective monoculture		No metabolites in the coculture		
Mt(2.1.0)**	Partial metabolic dominance with new metabolites	New metabolites unfound in the monoculture. Winner metabolites produced but some of them biotransformed		No metabolites in the coculture		Metabolism of the loser is completely blocked but the loser biotransformed some metabolites into its less toxic derivatives
Mt(2.0.1)**		New metabolites compared to the monoculture. Winner metabolite repertoire is enriched with compounds hardly found in the respective monoculture		No metabolites in the coculture		The loser activated some pathways of the winner
Mt(3.0.0)	Metabolic draw	Metabolites of both species present either at the same levels…				Both species retained their metabolic activity
Mt(3.1.0)***		… at the higher levels or…				
Mt(3.0.1)***		… at the lower levels compared to the monoculture				

^*^Denoted as Mt(1.1.1) if both cases take place at the same time

^**^Denoted as Mt(2.1.1) if both cases take place at the same time

^***^Denoted as Mt(3.1.1) if both cases take place at the same time

Upon these *dominances* and *dominance patterns* the *levels of dominance* were proposed and denoted as W-Y, where W is the symbol of the winning microbe and Y is the determined level of dominance (Table 2).

**Table 2:** Determination of *the level of dominance* in a two-species coculture upon Table 1

Dominances allowed	Level of dominance	Name of the level of dominance
K(1), M(1.0.0) and Mt(1.0.0) only	W-1	Total dominance
K(1), M(1.y.z) and Mt(1.y.z)	W-2	Partial dominance
K(1), M(1.y.z), Mt(1.y.z) and Mt(2.t.n)	W-3	
Either K(3) or M(3) present	W-4	
Either K(3) or M(3) with obligatory Mt(3) present	W:W-5	No dominance (draw)

**Fig. 1:**
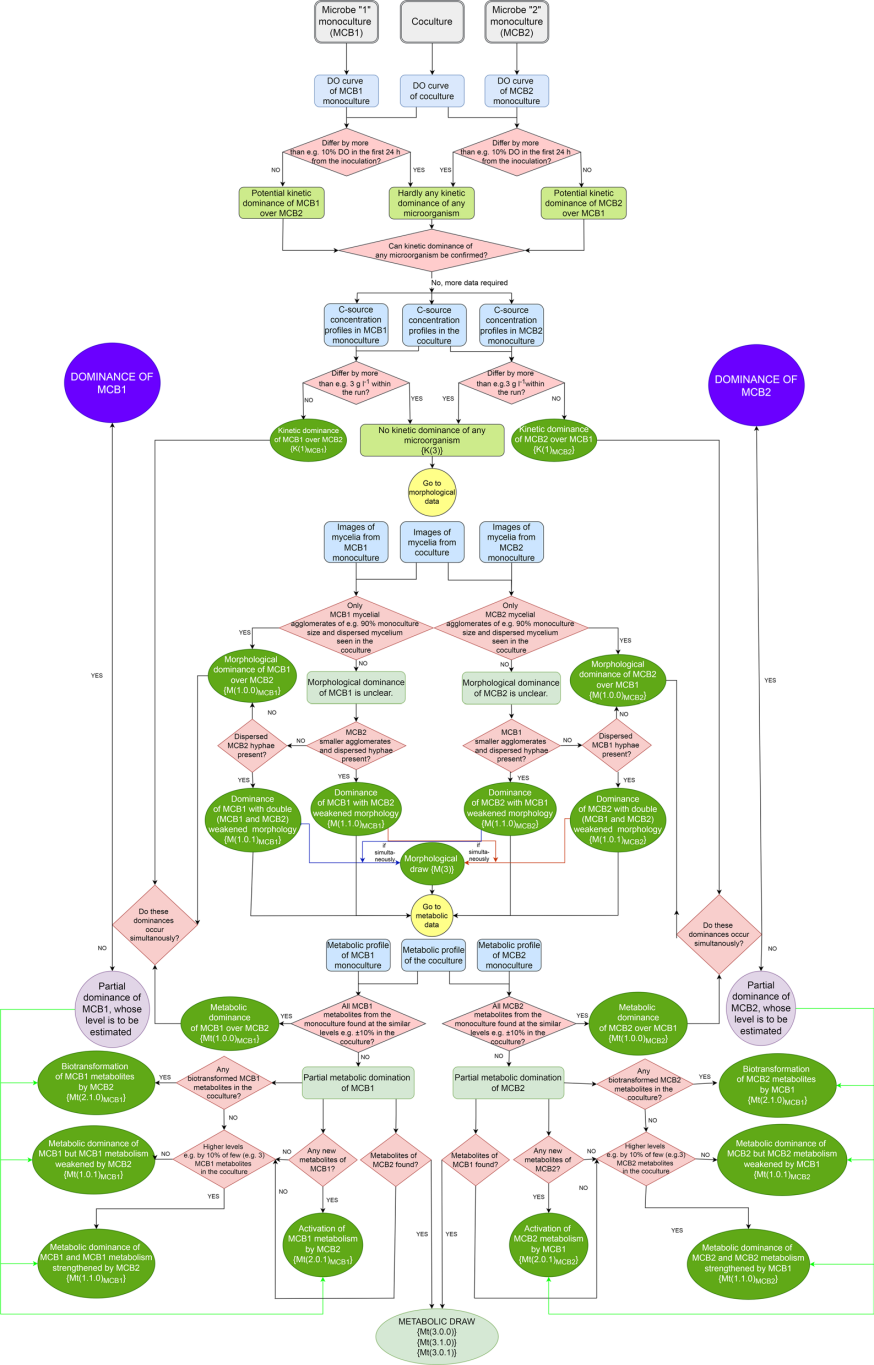
Determination of the outcome of the bioreactor cocultures with two competing species; DO stands for dissolved oxygen and C-source stands for carbon source

In Fig. 1 the algorithmic scheme is presented to evaluate the winner of the microbial competition in the coculture bioreactor. The arbitrary values (like “3 g l^−1^” or “90%” or “10%”) to show the difference between the monoculture and coculture curves for all types of data were suggested, however they must be treated as exemplary values only. The level to which the data from the coculture differ from the respective monoculture either MCB1 or MCB2 ones must be individually established on the basis of the known replicability of the experiments, entire statistical elaboration of the experimental data and general expertise on the studied microbial system. Similarly, the interpretation of the underlined comparative adjectives like “higher” or “lower” in Table 1 must be made.

In order to establish the kinetic dominance the use of two types of data is required. It is the common knowledge that during the cultivations run in bioreactors the oxygen level data are prone to high errors especially in the later stages of the process due to surface growth of microbes. Here one proposes to utilise oxygen profiles from the early hours of the process before these adverse effects take place. But if oxygen saturation data are still difficult to interpret, one may omit them and evaluate kinetic domination upon carbon substrate profiles only.

If the determination of the coculture outcome is made for the microorganisms whose cells do not differentiate, unlike filamentous fungi or actinomycetes, the determination of morphological parameters and consequently morphological domination can be omitted.

### Cases to illustrate various aspects of dominance

The proposed approach requires the experimental data from the coculture bioreactor with two species cultivated and two respective parallel monoculture bioreactors with these species growing as axenic cultures.

In order to experimentally support the proposed approach to determine the level dominance of a microorganism in the coculture, all the aspects of dominance and their outcomes described in Table 1 were exemplified in the form of cases supported by the experimental data from 25 bioreactor experiments (three bioreactors in each one: coculture and two monocultures).

In this section the experimental data came from the bioreactor runs which were simultaneously inoculated with spores or precultures of the respective filamentous microorganisms. For the sake of clarity the symbols and conditions of the experiments from which all below presented data were obtained are shown in Supplementary Tables from S1 to S4.

#### Case of kinetic dominance in the simultaneously inoculated cocultures

Growth kinetics seems to be the most important factor that determines the dominance of the individual species in accordance with the rule: the faster growing microbe defeats the slower growing one. This rule was not always satisfied and the quantitative determination of growth kinetics remains a certain challenge. In the analysis of the coculture it is difficult to use the measure that directly determines the amount of biomass growth, like dry weight measurement. It is impossible to separate two types of biomass present in the bioreactor. There are only indirect methods like microscopic analysis of the object by either light microscope or fluorescence one. The latter would require genetic modifications of the microbes to incorporate fluorescent protein marker in their genome. That is why oxygen utilization curves and carbon substrate concentration profiles were used as easily obtainable bioreactor run data.

**Fig. 2:**
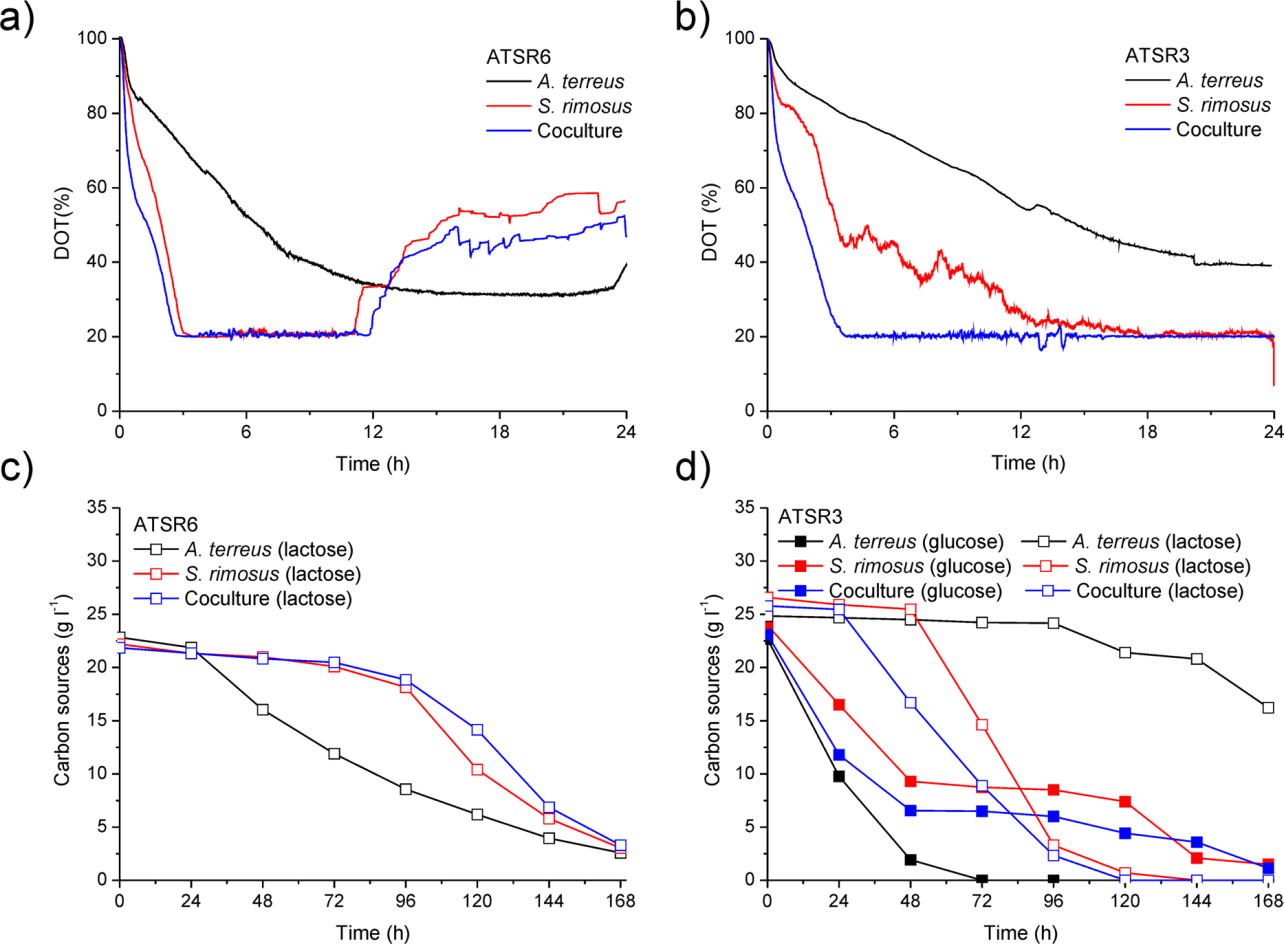
Kinetic data showing the competition between *A. terreus* and *S. rimosus* with the fungal winner (**a**) and (**c**) and draw (**b**) and (**d**); upon selected data from Boruta et al. (2021)

In Fig. 2 the determination of the kinetic dominance upon dissolved oxygen (DO) curves and carbon concentration curves is exemplified by the data for two various coculture experiments with competing *A. terreus* and *S. rimosus*. In Fig. 2a and c it is clearly seen that red (representing *S. rimosus* monoculture) and blue (representing coculture) curves are very close to each other what led to the conclusion that the kinetic dominance K(1) of *S. rimosus* over *A. terreus* took place. Otherwise, in Fig. 1b and d such conclusion could not be justified although blue curves seem to lie closer to red ones than to black ones but any unequivocal conclusion could not be drawn. C-source curve indicated on the dominance of *S. rimosus* but in our opinion it was not enough to draw the ultimate conclusion about its kinetic dominance K(3). That is why one can never decide about the winning microorganism only upon kinetic data. By the way later on *S. rimosus* occurred to be the partially dominating microorganism in the coculture in the metabolic aspect and ultimate winner of the experiment shown in Fig. 2b and d despite the lack of the kinetic dominance (Suppl. Fig. S1). The curves of *S. rimosus* metabolites in the coculture followed or even their levels were higher than those in *S. rimosus* monoculture and no *A. terreus* metabolites were detected in the coculture (Suppl. Fig. S1) (Boruta et al. 2021).

#### Case of morphological dominance in the simultaneously inoculated cocultures

**Fig. 3:**
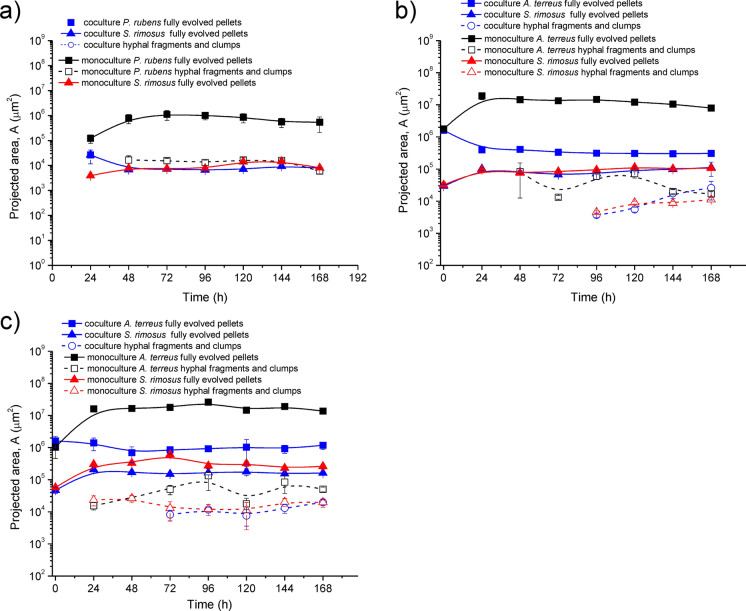
Cases of (**a**) morphological dominance of *S. rimosus* in PRSR1 (**b**) single-weakened morphology with winning *S. rimosus* in ATSR6 and (**c**) double-weakened morphology with winning *S. rimosus* in ASTR3; upon selected data from Boruta et al. (2021) and (2023a)

At first it must be mentioned that determination of morphological dominance was the most difficult task, mainly due to the high variability of the morphology during the development of filamentous species in the bioreactors and stochastic processes influencing this development. That is why the proposed aspects of morphological dominance in Table 1 are simplified to the highest extent. Frankly speaking, only morphological dominance M(1.0.0) can be determined without any doubts as the lack of agglomerates of one of the species, the entire loser, taking part in the competition is easily observable and measurable. All in all, in Fig. 3 three levels of morphological dominance are depicted in the forms of graphs showing the temporal changes of projected area of fungal (*A. terreus, P. rubens*) and actinomycete *S. rimosus* pellets.

**Fig. 4:**
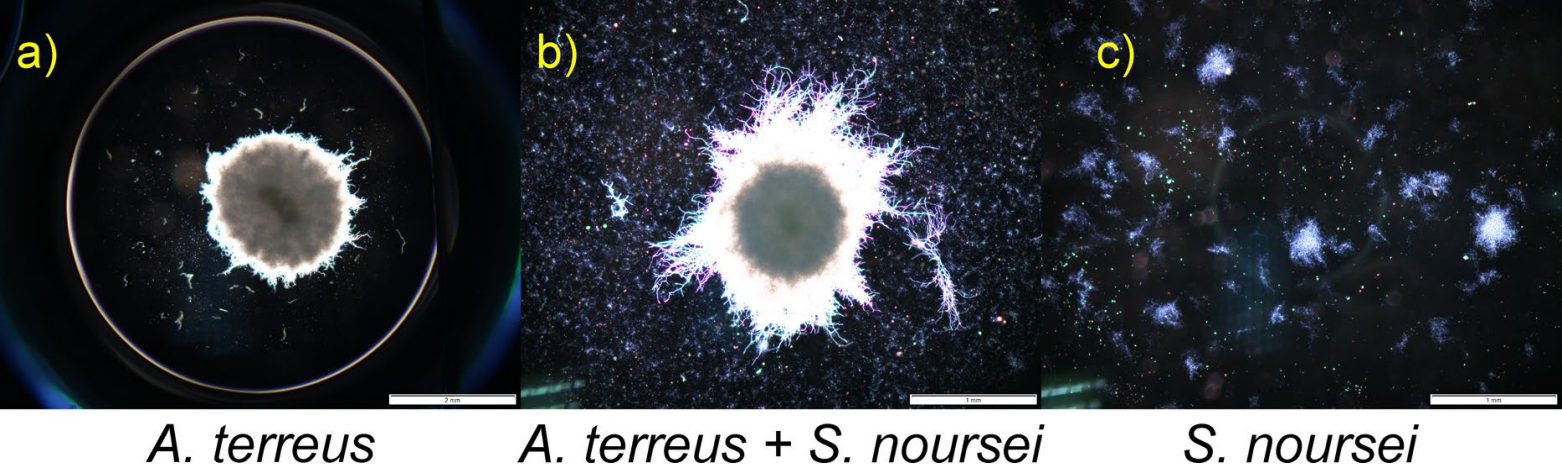
Exemplification of morphological draw in the competition of *A. terreus* and *S. noursei* determined by the comparison of *A. terreus* monoculture (**a**), coculture (**b**) and *S. noursei* monoculture (**c**); upon selected data from Boruta et al. (2023b)

In Fig. 3a there are no data to show the size of *P. rubens* pellets in the coculture. They simply did not exist. Only a few free small hyphal fragments and clumps were observed. It clearly exemplifies morphological dominance of *S. rimosus* over *P. rubens* (Boruta et al. 2023a). In Fig. 3b the case of weakened morphology M(1.1.0) of *A. terreus* competing with *S. rimosus* is shown. The size of *S. rimosus* pellets was the same both in the coculture and respective monoculture while fungal pellets were smaller than those in the monoculture (Ścigaczewska et al. 2021). Figure 3c exemplifies double weakened morphology M(1.0.1), in which both pellets of *A. terreus* and *S. rimosus* are smaller than their respective counterparts in the monocultures. In both experiments in which both types of weakened morphology were observed small hyphal fragment and clumps of both species were also detected (Ścigaczewska et al. 2021). In Fig. 4 the morphological draw is shown in the form of hyphal images. However, it must be remembered that it is the most difficult to unequivocally distinguish morphological draw from other types of morphological dominance due to the high variability of filamentous morphology (Boruta et al. 2023b).

#### Cases of metabolic dominance in the simultaneously inoculated cocultures

The analysis of the metabolic repertoires of the two microorganisms competing with each other in the coculture has to be made with the use mass spectrometry techniques. The detected ions of plausible secondary metabolites are next sought in the databases of natural compounds as described previously (Boruta et al. 2021, 2022, 2023a, b). Supported by the kinetic and morphological premises the determination of the metabolic repertoire ultimately brings the strongest and definitive evidence to indicate, which microorganism dominates. Furthermore, the potential to biosynthesize a variety of useful metabolites is the most desired information for any coculture. However, there is no need to find all metabolites and substances present in the cultivation broth. In the exemplary data presented further to show the dominances the number of metabolites found in various experiments was different depending on their number formed by the given microbial species and chance to putatively identify them. In spite of this, the use of the systematic approach to find the metabolic dominance is not excluded as one compares the coculture with the simultaneous monocultures and even two or three key metabolites may occur sufficient to determine the level of dominance.

The comparison of the coculture broth in relation to the respective monoculture broth may lead to different final outcomes (Table 1) that can be classified as metabolic dominance Mt(1.0.0) or partial metabolic dominance of one of the species within the range of various partial dominances. The presence of a metabolite or metabolites or its or their level(s) is a premise to classify the given case to one of the following group. The following cases were proposed to be distinguished (1) strengthened Mt(1.1.0) or weakened Mt(1.0.1) metabolism of the dominating microorganism (2) defence against the counterpart Mt(2.1.0), (3) activation of unveiled metabolic pathways of the dominating microorganism Mt(2.0.1) and (4) metabolic draw revealed in the presence of the metabolites of both counterparts like Mt(3.0.0), Mt(3.1.0) and Mt(3.0.1).

##### Metabolic dominance

The case of metabolic dominance should be understood as the situation in which one of the species completely blocks secondary metabolism of another one. In the case of metabolic dominance the levels of the metabolites are almost the same in the coculture and corresponding monoculture of the dominating microorganism. This aspect of dominance Mt(1.0.0) was experimentally exemplified by *P. rubens* and *S. rimosus* cocultures (Boruta et al. 2023a).

**Fig. 5:**
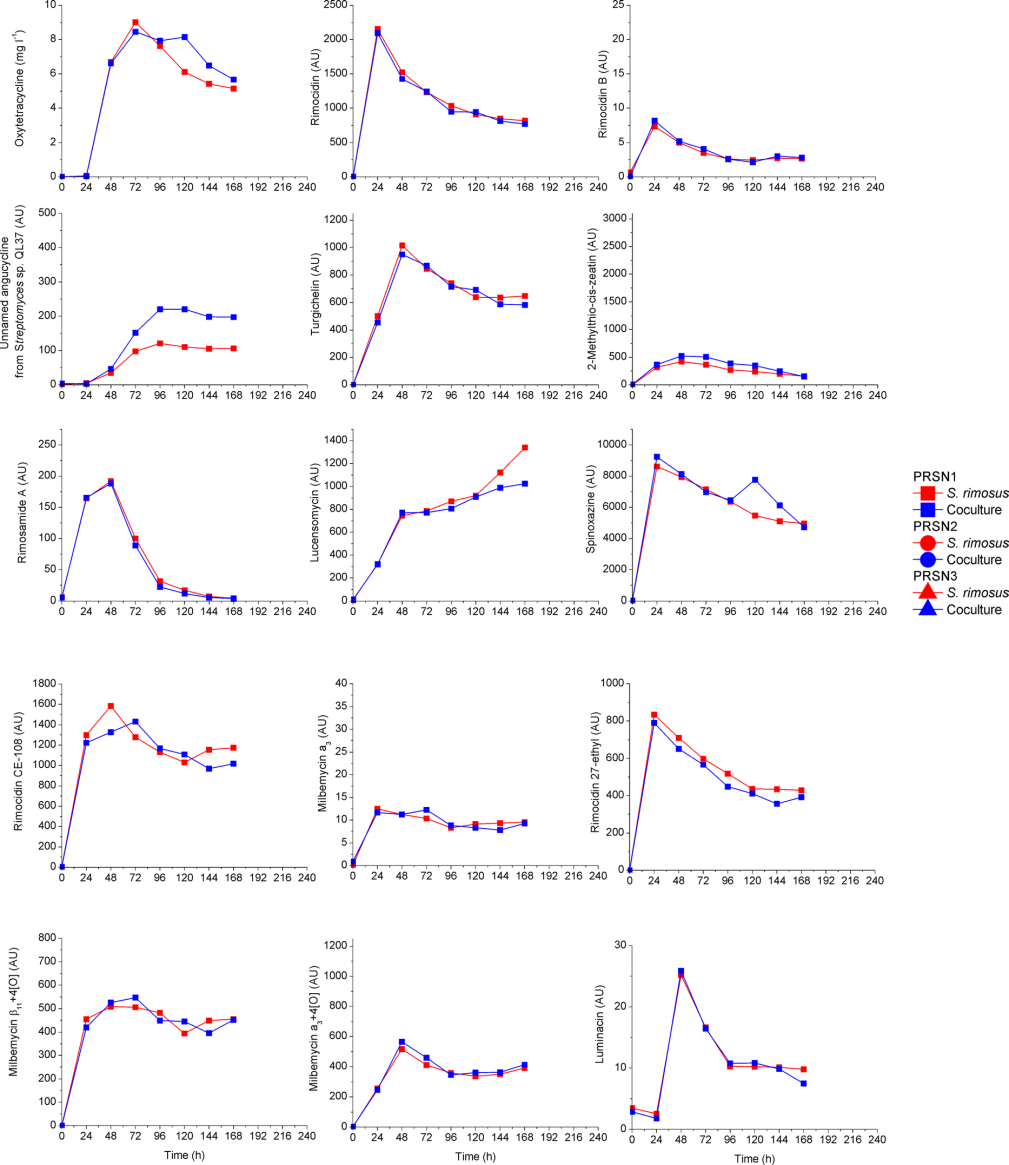
The case of metabolic dominance of *S. rimosus* over *P. rubens* expressed by the levels of sixteen *S. rimosus* metabolites in the coculture compared to the respective *S. rimosus* monoculture; upon selected data from Boruta et al. (2023a)

The temporal changes of the signals for fifteen metabolites of *S. rimosus* were practically identical in the coculture of *P. rubens* and *S. rimosus* and respective *S. rimosus* monoculture (Fig. 5). No *P. rubens* metabolites were detected in the coculture at all. It meant that actually *P. rubens* had shown no activity as even the levels of *S. rimosus* metabolites were hardly either increased or decreased due to any counteraction of the fungus. In this run also kinetic dominance was observed. Dissolved oxygen curves were identical for *S. rimosus* monoculture and co-culture in the first 48 h and so were the temporary changes of glucose and lactose concentration (Suppl. Fig. S2). In this run the morphological dominance was observed too (Fig. 3a).

##### Partial metabolic dominance

If metabolic dominance does not take place in the coculture, actually it is an infrequent case what can be concluded upon the studied literature (Boruta et al. 2021, 2022, 2023a, b) that a microorganism had practically no effect on its counterpart in the bioreactor cocultivation, then the cases of partial dominance should be considered. Herein few possibilities are described upon the comparative analysis of the sets of metabolites detected in the coculture and respective monocultures.

Partial metabolic dominance occurs in two variants, either some metabolites of the winning organism are produced at higher number and/or amounts than in the respective monoculture Mt(1.0.1) or some metabolites of the winning organism, are produced at lower number or/and amounts than in the respective monoculture Mt(1.1.0).

**Fig. 6:**
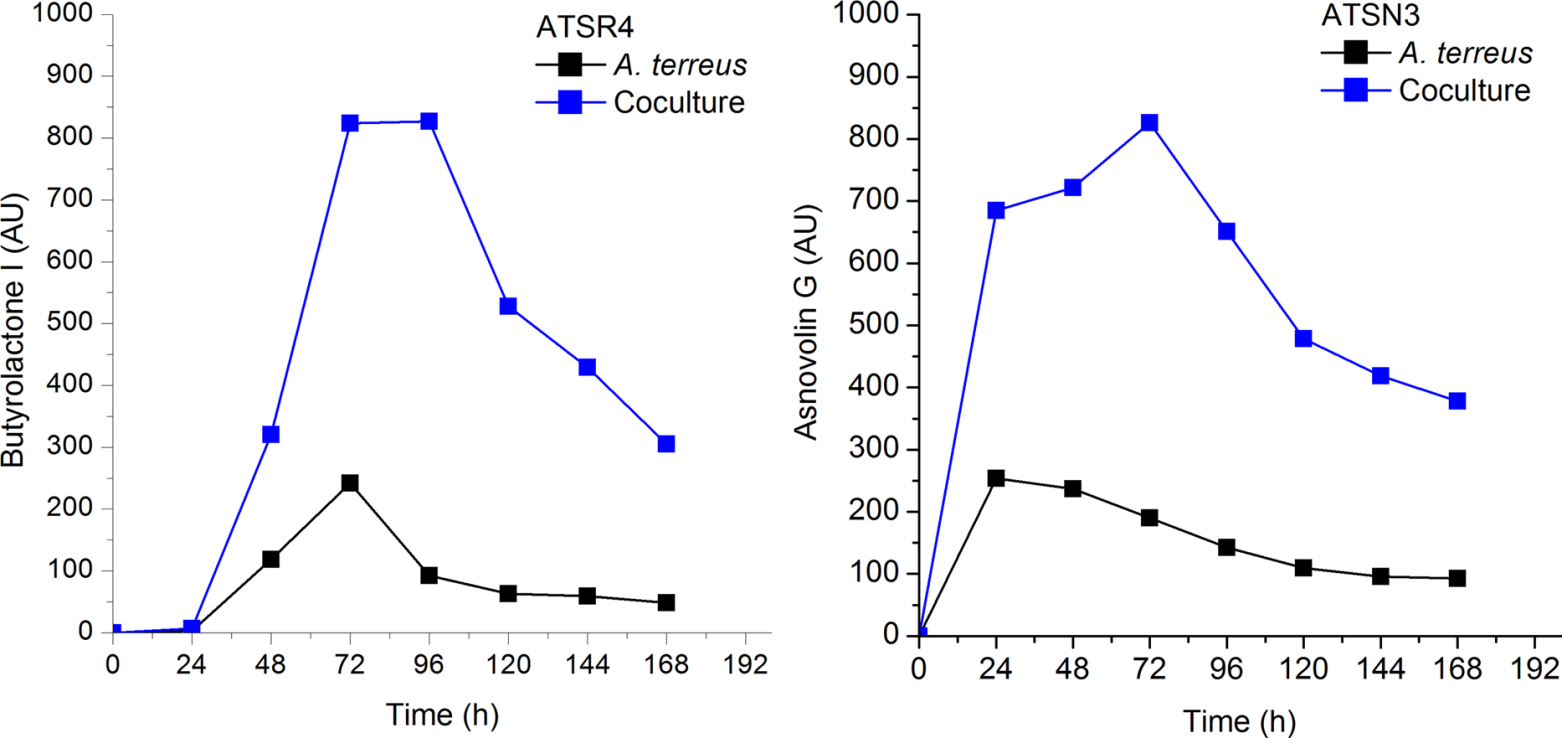
The case of partial metabolic dominance Mt(1.0.1) of *A. terreus* over *S. noursei*; upon selected data from Boruta et al. (2022)

**Fig. 7:**
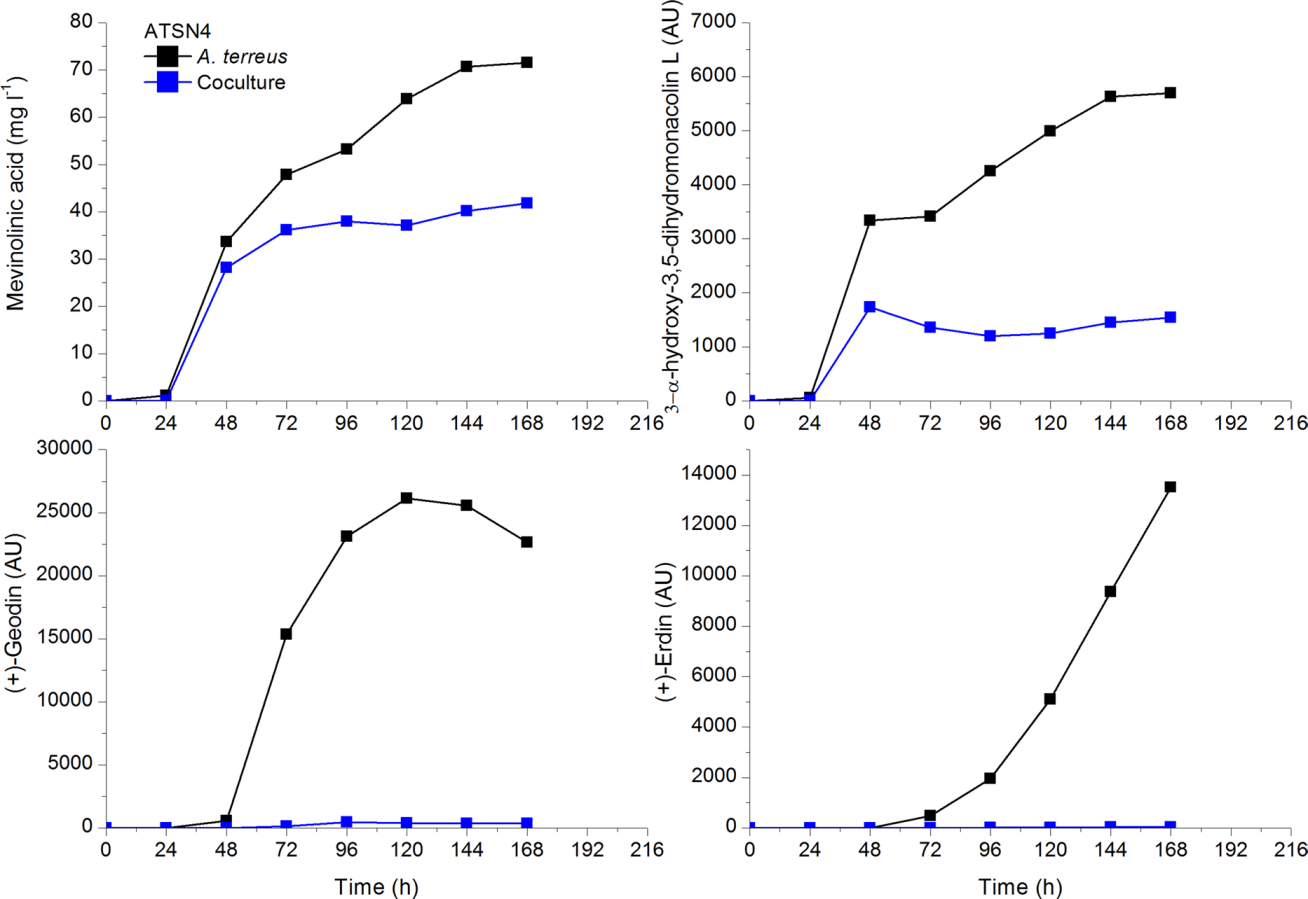
Blocked production of *A. terreus* octaketide metabolites ( +)-geodin and ( +)-erdin in the competition between *A. terreus* and *S. noursei* in ATSN4 run metabolically won by the fungus; nonaketide mevinolinic acid and its derivatives were produced at lower levels; upon selected data from Boruta et al. (2022)

In Fig. 6 the increased levels, compared to the respective monocultures, of two metabolites butyrolactone I and asnovolin G produced by the winning *A. terreus* which competed with *S. noursei* exemplify the pure case of partial metabolic dominance Mt(1.0.1) (Boruta et al. 2022). Furthermore, it is seen in Fig. 7 that *A. terreus*, the winner of the competition with *S. noursei*, had its octaketide metabolites of ( +)-geodin family production blocked, while for example its main secondary metabolite mevinolinic acid (lovastatin) was produced but at lower amounts. It exemplifies the pure case of partial metabolic dominance Mt(1.1.0).

It must be noticed that often both variants of partial metabolic dominance take place, like in the run denoted as ATSN4. In Fig. 6 butyrolactone I level is elevated while in Fig. 7 no octaketide production is observed in the same experiment. That is why many runs can be denoted as Mt(1.1.1) in accordance with Table 1.

##### Partial metabolic dominance with new metabolites

Partial metabolic dominance with new metabolites also takes place in the coculture in two variants. It is either biotransformation of the metabolites produced by the winner Mt(2.1.0) or enrichment of the metabolic repertoire of the winner microorganism Mt(2.0.1). In both cases it is the loser microorganism that contributes to the formation of these new metabolites. It is the feature of this type of dominance that the sought metabolites of interest are present in the coculture only. They are undetectable in the respective monoculture. The reason of the presence of biotransformed metabolites is probably connected with the struggle of the loser against its winning counterpart, while the completely new metabolites absent in the monoculture might be the effect of the activation of unveiled metabolic pathways (Boruta et al. 2021).

**Fig. 8:**
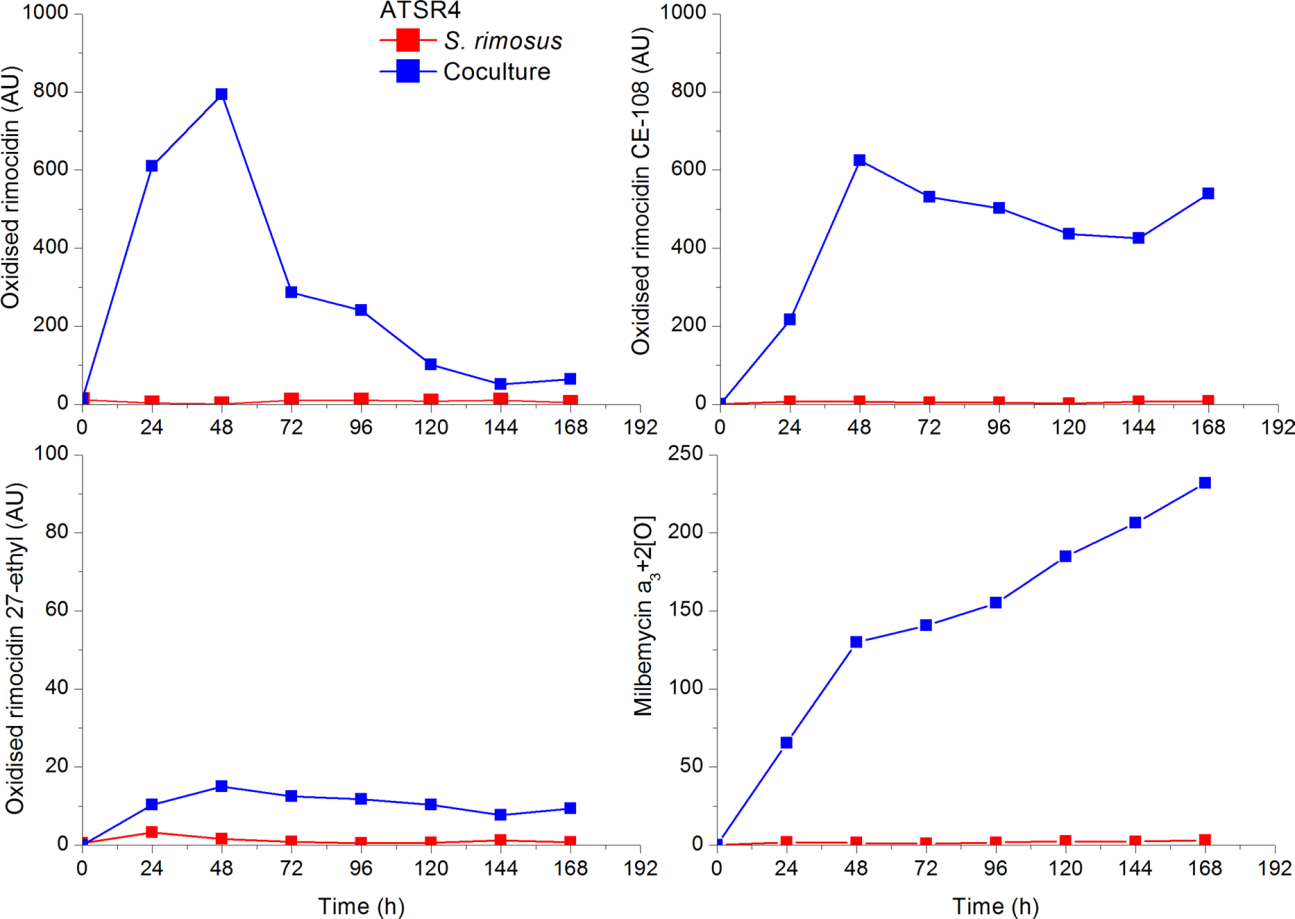
Levels of specifically modified metabolites of *S. rimosus* due to the action of *A. terreus* in the coculture experiments to exemplify partial metabolic dominance with transformed metabolites of the winning microorganism; upon selected data from Boruta et al. (2021)

*S. rimosus* is known to produce three types of rimocidins, namely rimocidin, rimocidin CE-108 and rimocidin with 27-ethyl group and milbemycin a_3_. However the analysis of chromatograms revealed the presence of unmentioned in any database metabolites whose *m/z* masses were somewhat related to the known metabolites. They occurred to be decarboxylated derivatives of all three rimocidins and specifically oxidised milbemycin a_3_. Never were these metabolites observed in *S. rimosus* monocultures but in the cocultures only (Fig. 8).

**Fig. 9:**
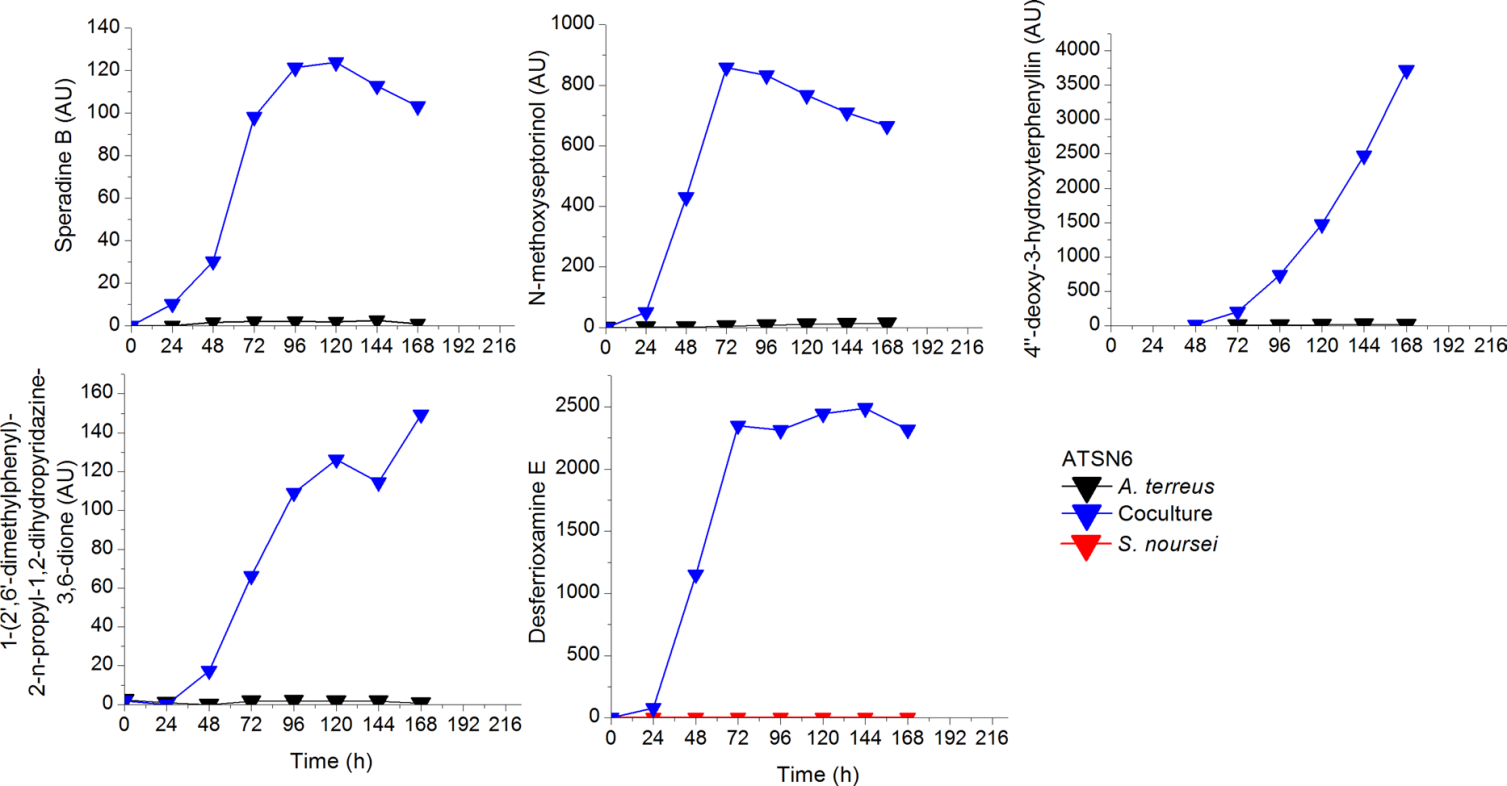
Activation of metabolic pathways of both species in ATSN6; upon selected data from Boruta et al. (2022)

It was *A. terreus* that must have destroyed the molecules of rimocidins being antifungal metabolites and modified milbemycin a_3_ (Boruta et al. 2021). Activation of unveiled metabolic pathways can be exemplified upon the competition of *A. terreus* and *S. noursei* (Boruta et al. 2022). Interestingly the activation of metabolic pathways in the coculture simultaneously inoculated by either spores or precultures was only observed in the run that finally ended with the draw, which is going to be exemplified in the next subsection and newly biosynthesised metabolites were produced by both species. However in that case more new metabolites were produced by *A. terreus* (Fig. 9). In Fig. 9 the presence of four *A. terreus* metabolites undetected in the monoculture is illustrated. These were speradine B, N-methoxyseptorinol, 4ʺdeoxy-3-hydroxyterphenyllin and 1-(2ʹ,6ʹ-dimethylphenyl)-2-n-propyl-1,2-dihydropyridazine-3,6-dione from *A. terreus* and desferrioxamine E from *S. noursei* (Boruta et al. 2022).

Eventually, it must be mentioned that partial metabolic dominance with new metabolites always accompanies the partial dominance of Mt(1.0.1) and Mt(1.0.1) type or metabolic draw like Mt(3.0.1).

##### Case of metabolic draw

The outcome of the cocultures in which metabolites of both species are observed is called a metabolic draw. Here it must be mentioned that the levels of the metabolites are of less interest as they can be lower than those in the respective monoculture or higher or even the formation of them can be strongly induced. That is why in accordance with Table 1 one has a metabolic draw of types Mt(3.0.0), Mt(3.1.0) or Mt(3.0.1).

**Fig. 10:**
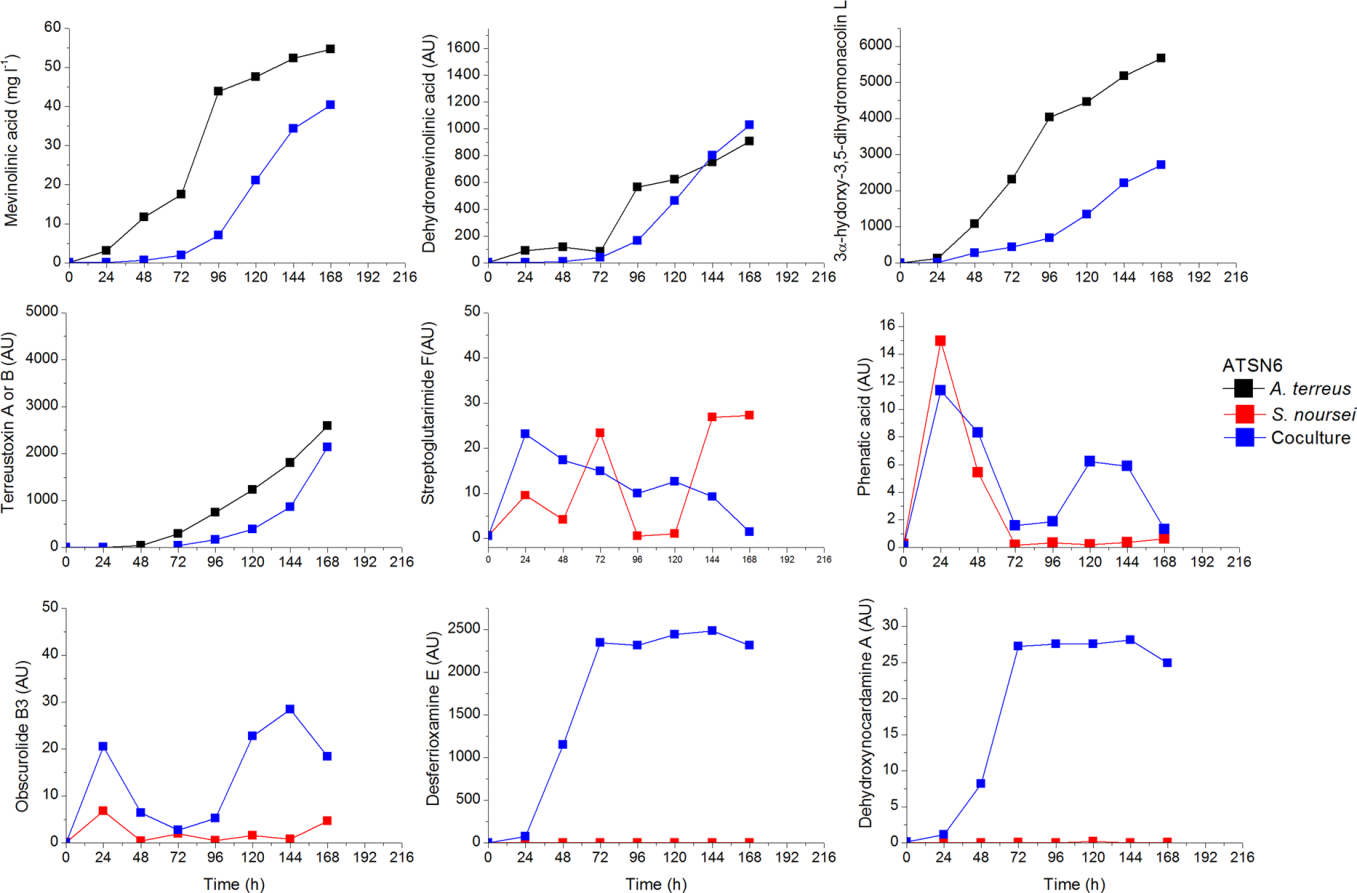
Simultaneous presence of metabolites from *A. terreus* and *S. noursei* in the coculture; upon selected data from Boruta et al. (2022)

The good example of metabolic draw is the aforementioned cocultivation of *A. terreus* and *S. noursei* described in the context of the activation of unveiled metabolic pathways. In Fig. 10 one can see a fairly rich collection of *A. terreus* and *S. noursei* metabolites simultaneously detected in the broth from the coculture bioreactor.

In order to sum up the examples of various levels of dominance Table 3 classifies the aforementioned literature cases of the simultaneously inoculated bioreactor cocultivations.

**Table 3:** The results of the competition between two species in the simultaneously inoculated cocultures

Experi-ment symbol	Competitors		Dominance pattern	Level of dominance	Literature
	Fungus (F)	Actinomycete (A)			
ATSR1	*A. terreus*	*S. rimosus*	{K(1)_A_, M(1.1.0)_A_, Mt(1.1.1)_A_, [Mt(2.1.0)_A_]} → (A)	A-3	Boruta et al. (2021)
ATSR2			{K(1)_A_, M(1.1.0)_A_, Mt(1.1.1)_A_, [Mt(2.1.0)_A_]} → (A)	A-3	
ATSR3			{K(3), M(1.0.1)_A_, Mt(1.1.1)_A_, [Mt(2.1.0)_A_]} → (A)	A-4	
ATSR4			{K(1)_A_, M(1.1.0)_A_, Mt(1.1.1)_A_, [Mt(2.1.0)_A_]} → (A)	A-3	
ATSR5			{K(1)_A_, M(1.1.0)_A_, Mt(1.1.1)_A_, [Mt(2.1.0)_A_]} → (A)	A-3	
ASTR6			{K(1)_A_, M(1.1.0)_A_, Mt(1.1.1)_A_, [Mt(2.1.0)_A_]} → (A)	A-3	
ATSR9			{K(1)_A_, M(1.1.0)_A_, Mt(1.1.1)_A_, [Mt(2.1.0)_A_]} → (A)	A-3	
ATSN3	*A. terreus*	*S. noursei*	{K(1)_F_, M(1.1.0)_F_, Mt(1.1.1)_F_} → (F)	F-2	Boruta et al. (2022)
ATSN4			{K(3), M(1.1.0)_F_, Mt(1.1.1)_F_} → (F)	F-4	
ATSN5			{K(1)_A_, M(1.1.0)_A_, Mt(1.1.1)_A_} → (A)	A-2	
ATSN6			{K(3), M(3), Mt(3.1.0), [Mt2.0.1_F_]} → (F:A)	F:A-5	
PRSR1	*P. rubens*	*S. rimosus*	{K(1)_A_, M(1.0.0)_A_, Mt(1.0.0)_A_} → (A)	A-1	Boruta et al. (2023a)
PRSN1	*P. rubens*	*S. noursei*	{K(1)_A_, M(1.0.0)_A_, Mt(1.0.0)_A_} → (A)	A-1	Boruta et al. (2023b)

### Strategy to change the winner of the microbial competition

Although the simultaneously inoculated runs of *A. terreus* and *S. rimosus* were conducted at different media compositions, none of them ended in the win of fungus (Boruta et al. 2021). In the similar *A. terreus* and *S. noursei* runs the only reason of more varied results was the fact that *S. noursei* could not utilise lactose that was a good source of carbon for *A. terreus* (Boruta et al. 2022). *Penicillium rubens* always lost when it competed with both actinomycetes studied (Boruta et al. 2023a, b). Medium composition, to be strict the type of carbon source, proved to be too weak strategy to drastically change the outcome of the cocultures.

Due to the fact that the elevated number of new metabolites or enhanced levels of other metabolites were observed in the runs that ended in draw or partial dominance the strategy to equalise the chances for the losing species was sought. It was realised by a more powerful strategy, namely the time shift (delayed) inoculation. In this method coculture bioreactor is inoculated either by spores or preculture with one microorganisms and the second microorganism is introduced to the coculture bioreactor after the selected period of time 24 or 48 h (Boruta et al. 2021, 2022, 2023a, b).

The outcomes of the delayed inoculation led to either the change of the winner or change of the dominance within time of the run process and these cases were described further.

#### Change of the aspects of dominance in the run with delayed inoculation

As claimed by Boruta et al*. S. rimosus* was too aggressive and it always subdued *A. terreus*. Only delayed inoculation let *A. terreus* win and activate the yet unveiled metabolites (Boruta et al. 2021).

Also *A. terreus* showed the activation of some metabolites in the won competition with *S. noursei*, although delayed inoculation was not obligatory here to make *A. terreus* a winner (Boruta et al. 2022). The results of the cases in which *A. terreus* inoculated with the delay competed with actinomycetes are collected in Table 4.

**Table 4:** The change of the dominance pattern induced by delayed inoculation for the experiments described in literature

Experi-ment symbol	Competitors		Dominance pattern	Level of domi-nance	Litera-ture
	Fungus (F)	Actinomycete (A)			
ATSR5*	*A. terreus* inoculated from preculture at t = 0 h	*S. rimosus* inoculated from preculture at t = 0 h	{K(1)_A_, M(1.1.0)_A_, Mt(1.1.1)_A_, [Mt(2.1.0)_A_])} → (A)	A-3	Boruta et al. (2021)
ATSR8*	*A. terreus* inoculated from preculture at t = 0 h	*S. rimosus* inoculated from preculture at t = 24 h	{K(1)_F_, M(1.0.0)_F_, Mt(1.1.1)_F_, [Mt(2.1.1)_F_])} → (F)	F-3	
ASTR6*	*A. terreus* inoculated from preculture at t = 0 h	*S. rimosus* inoculated from preculture at t = 0 h	{K(1)_A_, M(1.1.0)_A_, Mt(1.1.1)_A_, [Mt(2.1.0)_A_])} → (A)	A-3	
ATSR7*	*A. terreus* inoculated from preculture at t = 0 h	*S. rimosus* inoculated from preculture at t = 24 h	{K(1)_F_, M(1.0.0)_F_, Mt(1.1.1)_F_, [Mt(2.1.1)_F_])} → (F)	F-3	
ATSN5	*A. terreus* inoculated from preculture at t = 0 h	*S. noursei* inoculated from preculture at t = 0 h	{(K(1)_A_, M(1.1.0)_A_, Mt(1.1.1)_A_)} → (A)	A-2	Boruta et al. (2022)
ATSN7**	*A. terreus* inoculated from preculture at t = 24 h	*S. noursei* inoculated from preculture at t = 0 h	{(K(1)_A_, M(1.1.0)_A_, Mt(1.1.1)_A_)} → (A)	A-2	
ATSN8	*A. terreus* inoculated from preculture at t = 0 h	*S. noursei* inoculated from preculture at t = 24 h	{(K(1)_F_, M(1.0.0)_F_, Mt(1.1.1)_F_)} → (F)	F-2	

^*^Respective ASTR5 and ATSR8 runs differed from respective ASTR6 and ATSR7 runs by carbon source composition respectively glucose and lactose, and glucose (details in Table S1)

^**^In this case the winner did not change as *A. terreus* was inoculated into *S. noursei* developing for 24 h

**Fig. 11:**
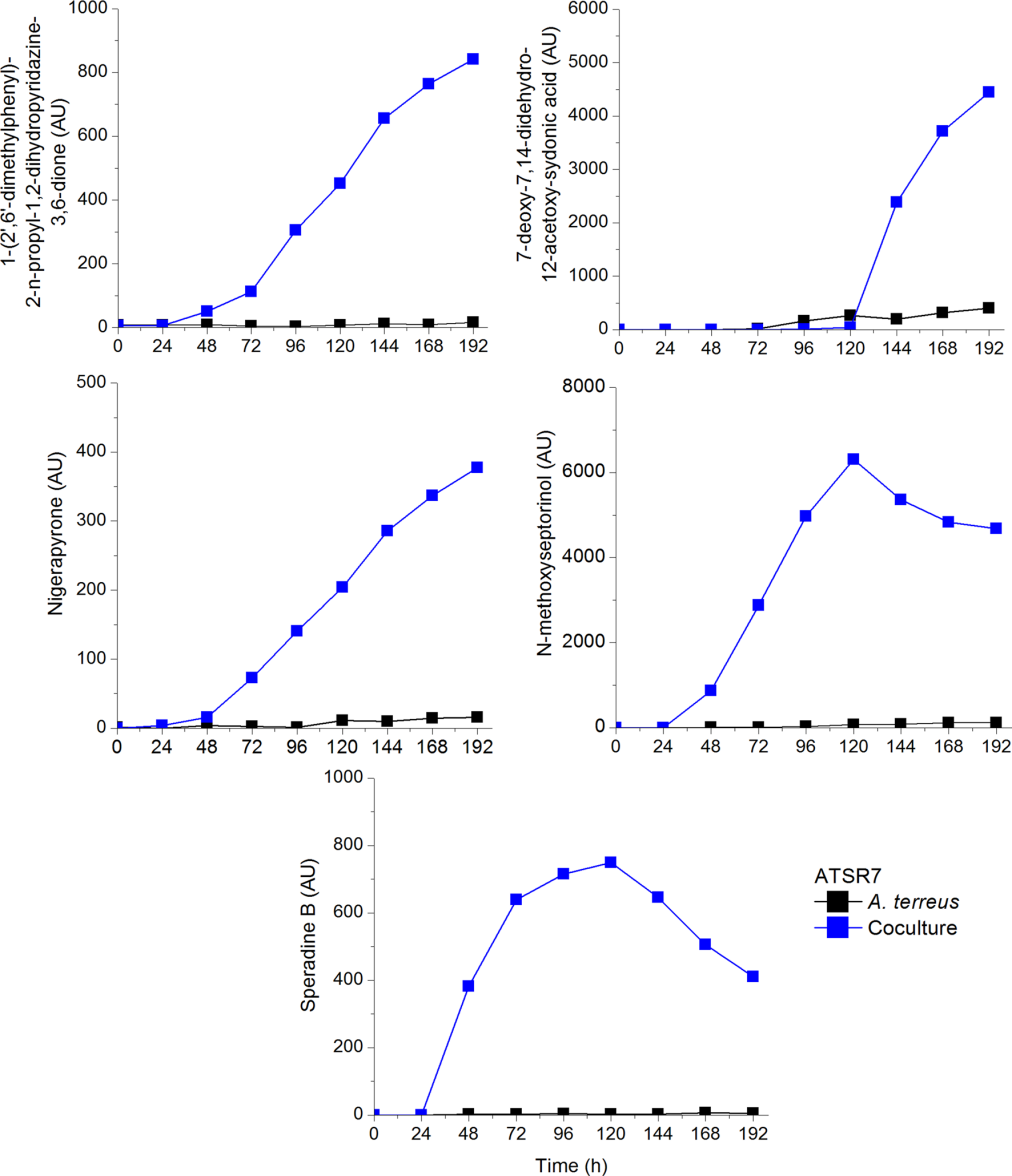
Activation of unveiled in the monoculture metabolic pathways of *A. terreus* in the won competition with *S. rimosus*; inoculation of *S. rimosus* preculture was delayed by 24 h; upon selected data from Boruta et al. (2021)

The Mt (2.1.1)_F_ effect in the *A. terreus* and *S. rimosus* competition eventually won by the fungus due to the delayed inoculation is exemplified in Fig. 11. Four metabolites coming from *A. terreus*, namely 1-(2ʹ,6ʹ-dimethylphenyl)-2-n-propyl-1,2-dihydropyridazine-3,6-dione, nigerapyrone, N-methoxyseptorinol, 7-deoxy-7,14-didehydro-12-acetosydonic acid were found in the coculture only (Boruta et al. 2021).

**Fig. 12:**
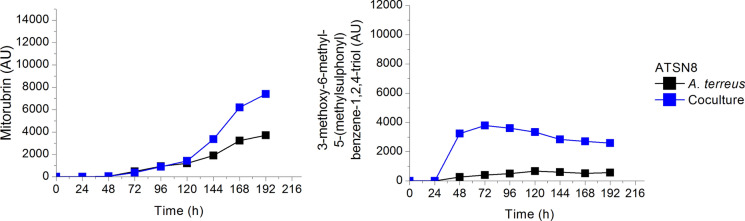
The example of increase of metabolite levels in the coculture of *A. terreus* in the won competition with *S. noursei;* inoculation of *S. noursei* preculture was delayed by 24 h; upon selected data from Boruta et al. (2022)

In Fig. 12 the elevated levels of two *A. terreus* metabolites mitorubrin and 3-methoxy-6-methyl-5-(methylsulphonyl)benzene-1,2,4-triol observed in the coculture exemplify the case of increased levels of metabolites of the winning microorganism *A. terreus* that defeated *S. noursei* in the delayed inoculation run.

#### Change of the aspects of dominance in the run with delayed inoculation—defeat of the initial winner within the duration of the process

**Fig. 13:**
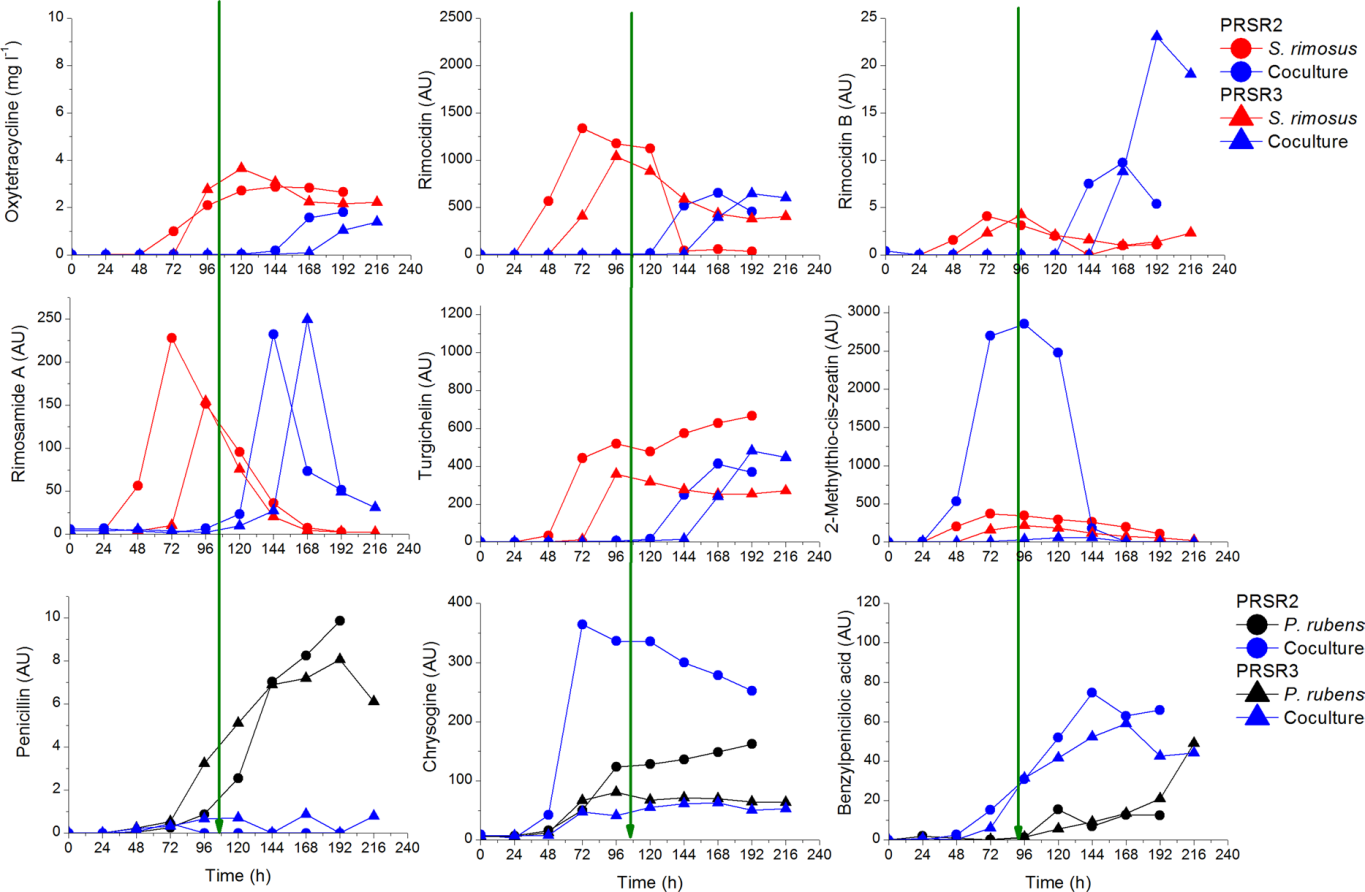
Profiles of metabolites in the two-species microbial competition in which the initial winner was ultimately led to the draw of green arrows denote approximate (± 12 h) time of change of dominance, namely revival of the actinomycete; upon selected data from Boruta et al. (2023a)

The competition of *P. rubens* with any actinomycete had always ended with the defeat of the fungus nevertheless the delayed inoculation gave initial kinetic, morphological and partial metabolic dominance to the fungus. Its metabolites were also produced but only the ones that normally occur in the early hours of the run, which was not the case of penicillin. Ultimately both *S. rimosus* and *S. noursei* stroke back and began to destroy the fungus. Actinomycete metabolites started to be produced in the coculture as shown by green arrows in Fig. 13, what exemplifies the end of *P. rubens* dominance. In the late hours of the runs both metabolites of *P. rubens* and *S. rimosus* were present in the coculture (Boruta et al. 2023a, b).

**Fig. 14:**
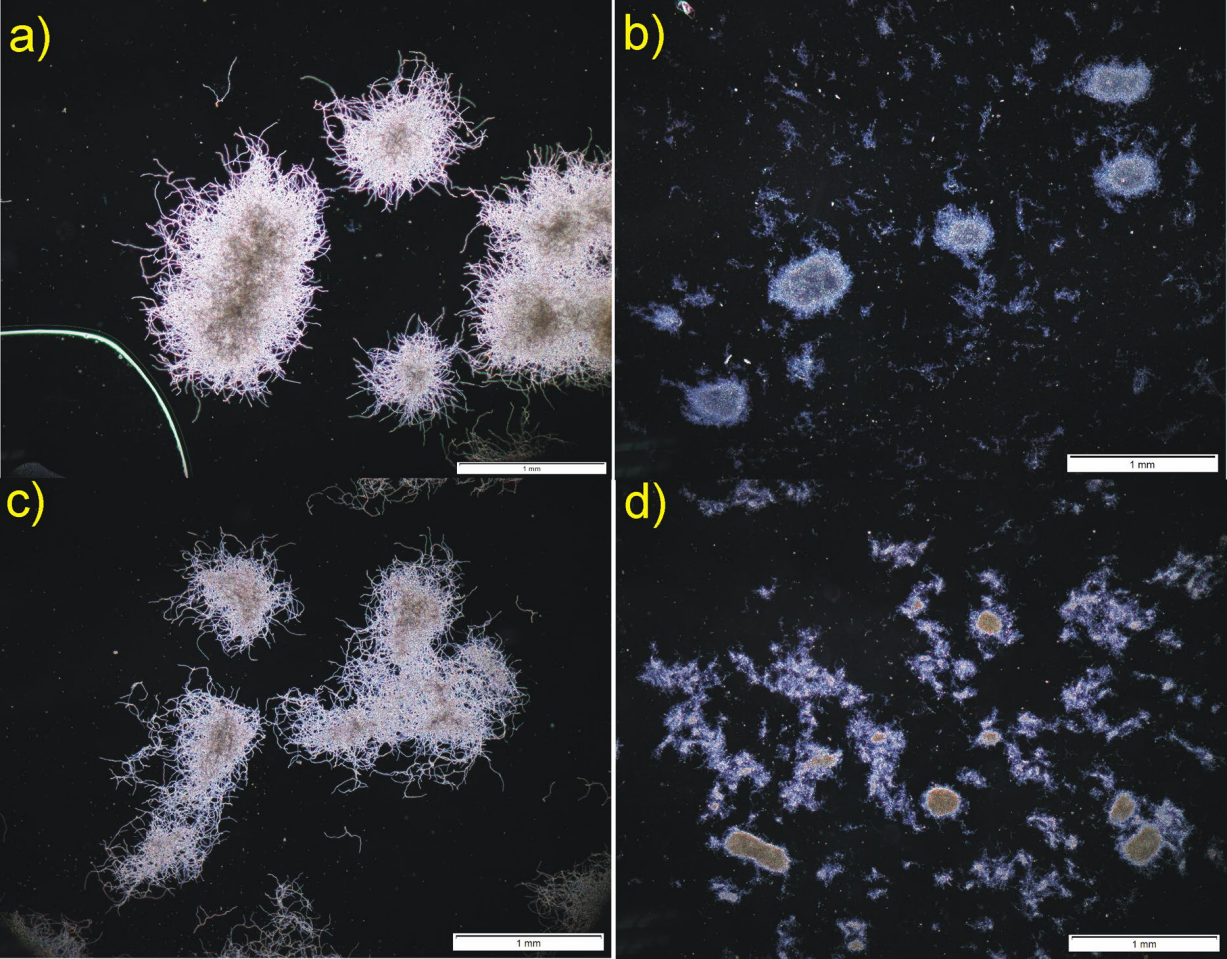
Dominating *P. rubens* (**a**) and *P. rubens* (**c**) experiencing morphological changes (deterioration) caused by the gradual revival of *S. rimosus* (**b**) and (**d**) in the coculture of these two microorganisms; upon selected data from Boruta et al. (2023a)

The aspect of morphological dominance well supplements the example of the overall change of dominance in the competition between *P. rubens* and *S. rimosus*. In Fig. 14a the pellets of *P. rubens* are seen at 48 h of the run, just 24 h after introducing *S. rimosus* spores. They morphologically dominated over *S. rimosus* whose spores had not germinated yet. But after 192 h *P. rubens* was definitely overwhelmed (Fig. 14b) and *S. rimosus* mycelium, although not in the form of strong pellets led to the draw in the competition. The similar action is seen in Fig. 14c and d for PRSR3 run but it was only moved in time as the introduction of *S. rimosus* took place at 48 h of the run.

All in all, despite the great advantage granted to *P. rubens* within the first half of the cultivation, actinomycete finally ended the competition in draw. In Table 5 the dominance patterns for the cases of delayed inoculation of both studied actinomycete into *P. rubens* are presented.

**Table 5:** The change of the dominance pattern within the time of the cocultivation induced by the delayed inoculation for the experiments described in literature

Experi-ment symbol	Competitors		Dominance pattern	Level of domi-nance	Litera-ture
	Fungus (F)	Actino-mycete (A)			
PRSR1*	*P. rubens*	*S. rimosus*	{K(1)_A_, M(1.0.0)_A_, Mt(1.0.0)_A_} → (A)	A-1	Boruta et al. (2023a)
PRSR2	*P. rubens* inoculated from spores at t = 0 h	*S. rimosus* inoculated from spores at t = 24 h	From 0 to 96 h {K(1)_F_, M(1.1.0)_F_, Mt(1.0.1)_F_} → (F)	A-2	
			From 96 h {K(3), M(1.0.1)_F_, Mt(3.0.1)} → (F:A)	F:A-5	
PRSR3	*P.rubens* inoculated from spores at t = 0 h	*S. rimosus* inoculated from preculture at t = 48 h	From 0 to 120 h {K(1)_F_, M(1.0.0)_F_, Mt(1.0.1)_F_} → (F)	A-2	
			From 120 h {K(3), M(1.0.1)_F_, Mt(3.0.1)} → (F:A)	F:A-5	
PRSN1*	*P. rubens*	*S. noursei*	{K(1)_A_, M(1.0.0)_A_, Mt(1.0.0)_A_} → (A)	A-1	Boruta et al. (2023b)
PRSN2	*P. rubens* inoculated from spores at t = 0 h	*S. noursei* inoculated from spores at t = 24 h	From 0 to 96 h {K(1)_F_, M(1.1.0)_F_, Mt(1.0.1)_F_} → (F)	A-2	
			From 96 h {K(3)_A_, M(1.0.1)_F_, Mt(3.0.1)_F_} → (F:A)	F:A-5	
PRSN3	*P. rubens* inoculated from spores at t = 0 h	*S. noursei* inoculated from spores at t = 48 h	From 0 to 120 h {K(1)_F_, M(1.0.0)_F_, Mt(1.0.1)_F_} → (A)	A-2	
			From 120 h {K(3)_A_, M(1.0.1)_F_, Mt(3.0.1)_F_} → (F:A)	F:A-5	

^*^Simultaneous inoculation cases presented as the reference

## Discussion

Cocultures are believed to be a method by which microorganisms produce new metabolites normally unavailable in the monocultures or increase levels of the metabolites produced in the monocultures. Using the general terms, in both cases one counts on the response of a microorganism to the presence of the other one. Only when new metabolites or increased level of the other ones were obtained the process could be considered as successful. That is why each time the accurate determination of the level of dominance of the competitors in the two-species coculture gives us the new knowledge about the outcome of the bioreactor run as it is the level of dominance that ultimately determines the final outcome of the cocultivation of two species.

The sense of seeking secondary metabolites in the coculture was justified by Bertrand et al. (2014). The profits from the cocultivation, including awakening of cryptic gene clusters and pathways aimed at drug discovery were considered in their review. Unfortunately, no submerged bioreactor co-cultures were reported then. Mittermeier et al. in their review showed the variety of literature examples of coculture among various species, nevertheless the secondary metabolism of any microorganism in the cocultures was not addressed (Mittermeier et al. 2023a).

Generally, there is a shortage of original scientific papers that would deal with microbial cocultures in the bioreactors. That is why it is hard to find the data to which the present study could be referred to, excluding the ones upon which the systematic approach was created (Boruta et al. 2021, 2022, 2023a, b; Ścigaczewska et al. 2021).

There are few examples that the bioreactor cocultures were applied for the biosynthesis of alcohols or organic acids like the production of ethanol and xylitol by *Saccharomyces cerevisiae* and *Spathaspora arborariae* (Hickert et al. 2013) or ethanol, 1-butanol and 1-hexanol from carbon monoxide by the synthetic co-culture of *Clostridia* (Bäumler et al. 2023). The filamentous microorganism co-culture was only represented by *Trichoderma reesei* and *Aspergillus* *niger* cultivated in a fed-batch stirred tank bioreactor to enhance the production of cellulases (Ahamed and Vermette 2008). Similarly, Mittermeier et al. (2023b) made experiments with the same microbial system in the batch bioreactors of 0.7 and 3.0 L volume. The data mentioned in this paragraph are actually the only coculture studies in the stirred tank bioreactors reported by other authors.

Regarding the studies of secondary metabolism in the cocultures, Sun et al. (2021) proved the induction of secondary metabolism of *Aspergillus sydowii* by *Bacillus subtilis* finding 25 metabolites but in the scale of agar plates.

Rateb et al. studied the coculture of a filamentous fungus *Aspergillus fumigatus* and actinomycete *Streptomyces bulli*, showing the effect of the induction of secondary metabolism in the scale of 1 L liquid medium but not in stirred tank bioreactors (Rateb et al. 2013). Only Luti and Mavituna (2011) proved the enhancement of *Streptomyces coelicolor* production of undecylprodigiosin in the coculture with *Escherichia coli* in a 2-L stirred tank bioreactor. At the same time actinorrhodin production was repressed.

The systematic approach presented above revealed that the enhancement of any metabolite formation did not take place too frequently. If the level of dominance of any microorganism is high, it is hardly possible to obtain the desired outcome of the process. It predominantly takes places when the inoculation of the bioreactor is simultaneous. The faster-growing or more (colloquially speaking) “aggressive” microorganism is always the winner over the weaker counterpart. A question arises what would happen if growth rates of two microbes were approximately equal to each other. It does not mean that the competition would end in draw. The aggressiveness of a microbe is not only the issue of its growth rate but also of antibiotic metabolites excreted to struggle against the counterpart. Furthermore, the medium composition influences the final outcome of the competition. As it is unlikely to compose the optimum common medium in the coculture bioreactor for both cultivated species, one of them may gain advantage for example in the case when a carbon source is hardly utilised by its counterpart. Thus the strategy of manipulating with medium composition may take the opportunity to change the ultimate outcome of the competition. In our studies such competition ended in the draw when the two microbes occurred to have similar levels of aggressiveness when the medium was not optimal for the theoretically more aggressive microorganism. It was the case of one coculture of *A. terreus* and *S. noursei*, in which the metabolites of both species were present. Upon all experimental data and systematic approach *S. noursei* was shown to be a less aggressive actinomycete with less lethal arsenal of metabolites against its counterparts compared to *S. rimosus*.

But only in the case of delayed inoculation, which is a far more powerful strategy to change the outcome of the coculture by means of taming the more aggressive species and giving advantage to a weaker organism, new metabolites may be formed like in the case of the competition of *A. terreus* and *S. rimosus*, which ended with the partial dominance of the fungus. Also in these cases the ultimate draw may take place, like for *P. rubens* (not so “aggressive” fungus) competing with the studied actinomycetes.

The findings resulting from our systematic approach occurred to be supported by the experimental data from the aforementioned bioreactor system with secondary metabolisms described by Luti and Mavituna (2011). The addition of *E. coli* was designed so that the growth of *S. coelicolor* was not repressed and *E. coli* played the role of elicitor. None of the species exerted the high level of dominance, what led to the increase of desired metabolite undecylprodigiosin.

To sum up, the proposed systematic approach can be in practice applied for any two species microbial systems, in which filamentous organisms grow and is not limited to *Aspergilli*, *Penicilli* or *Streptomyces* genera. In order to use it the experimental data on oxygen and substrate utilisation kinetics, hyphal morphology and secondary metabolites should be available. Furthermore, already upon kinetic and morphological data first premises on the level of dominance can be found to be ultimately confirmed by the metabolite data. There is no condition that one needs the complete metabolic repertoire, which is actually impossible to be determined. Even few key metabolites are sufficient. It makes the approach flexible and prone to the tuning by its user. If one or both microorganisms studied does not differentiate their cells the omission of the morphological dominance is allowable.

## Conclusions

On the basis of the study the following conclusions can be drawn.

- The systematic approach in the kinetic, morphological and metabolic aspects is required to determine the outcome of the cocultivation of two microbial species.
- The results of this analysis allow for the better choice of the process conditions including the method of inoculation for the future cocultivation so as to achieve the desired effect of metabolism enhancement and formation of new metabolites with as little number of experiments as possible.
- In order to activate new pathways or enhance the formation of already produced metabolites the competition between two microbes should end in the draw or the change of the dominating microorganism in the given pair of counterparts should take place. Only in few cases partial dominance may bring the desired result of the draw.
- Once the stronger microorganism with the tendency to dominate its counterpart in the coculture is determined, such measures as the change of media composition or delayed inoculation should be undertaken to promote the weaker microorganism.

## Supplementary Information

Below is the link to the electronic supplementary material.

Supplementary file1 (PDF 325 kb)

## Data Availability

All data supporting the findings of this study are available within the paper and its Supplementary Information.
