# Supplementary material for: A systematic approach to determine the outcome of the competition between two microbial species in bioreactor cocultures
Source: Antonie Van Leeuwenhoek. 2024 Nov 14;118(1):26. doi: 10.1007/s10482-024-02035-y (PMC11564368; doi:10.1007/s10482-024-02035-y)
Supplement: Supplementary file 1 — Supplementary file1 (PDF 325 kb) [file 10482_2024_2035_MOESM1_ESM.pdf]

Supplementary materials

A systematic approach to determine the outcome of the competition between two microbial species in bioreactor cocultures

Marcin Bizukojć*, Tomasz Boruta, Anna Ścigaczewska

Lodz University of Technology, Faculty of Process and Environmental Engineering, Department of Bioprocess Engineering, ul. Wólczańska 213, 93-005 Łódź, Poland; marcin.bizukojc@p.lodz.pl

* corresponding author

Suppl. Table S1 Compositions of the cultivation media used in the ATSR experiments (Boruta et al. 2021)

Medium component	Experiment								
	ATSR1	ATSR2	ATSR3	ATSR4	ATSR5	ATSR6	ATSR7	ATSR8	ATSR9
Glucose (g l^{-1})	20	20	20	20	20	0	0	20	0
Lactose (g l^{-1})	0	0	20	20	20	20	20	20	20
Yeast extract (g l^{-1})	5	5	2	4	4	4	4	4	4
$(NH_4)_2SO_4$ (g l^{-1})	0	2	2	0	0	0	0	0	0
KH_2PO_4 (g l^{-1})	1.51	1.51	1.51	1.51	1.51	1.51	1.51	1.51	1.51
NaCl (g l^{-1})	0.4	0.4	0.4	0.4	0.4	0.4	0.4	0.4	0.4
$MgSO_4 \cdot 7H_2O$ (g l^{-1})	0.5	0.5	0.5	0.5	0.5	0.5	0.5	0.5	0.5
Biotin (mg l^{-1})	0.04	0.04	0.04	0.04	0.04	0.04	0.04	0.04	0.04
Trace element solution (ml l^{-1})*	1	1	1	1	1	1	1	1	1

* Trace elements solution of the following composition: $MnSO_4$ 50 mg l^{-1}, $ZnSO_4 \cdot 7H_2O$ 1 g l^{-1}, $Fe(NO_3)_3 \cdot 9H_2O$ 2 g l^{-1}, $Na_2B_4O_7 \cdot 10H_2O$ 100 mg l^{-1}, $CuSO_4 \cdot 5H_2O$ 250 mg l^{-1} and $Na_2MoO_4 \cdot 2H_2O$ 50 mg l^{-1}.

Suppl. Table S2 Compositions of the cultivation media used in the ATSN experiments (Boruta et al. 2022)

Medium component	Experiment								
	ATSN3	ATSN4	ATSN5	ATSN6	ATSN7	ATSN8			
Glucose (g l^{-1})	0	20	20	0	20	20			
Lactose (g l^{-1})	20	20	20	20	20	20			
Yeast extract (g l^{-1})	4	4	4	4	4	4			
$(NH_4)_2SO_4$ (g l^{-1})	0	2	2	0	0	0			
KH_2PO_4 (g l^{-1})	1.51	1.51	1.51	1.51	1.51	1.51			
NaCl (g l^{-1})	0.4	0.4	0.4	0.4	0.4	0.4			
$MgSO_4 \cdot 7H_2O$ (g l^{-1})	0.5	0.5	0.5	0.5	0.5	0.5			
Biotin (mg l^{-1})	0.04	0.04	0.04	0.04	0.04	0.04			
Trace element solution (ml l^{-1})*	1	1	1	1	1	1			

* Trace element solution of the following composition: $MnSO_4$ 50 mg l^{-1}, $ZnSO_4 \cdot 7H_2O$ 1 g l^{-1}, $Fe(NO_3)_3 \cdot 9H_2O$ 2 g l^{-1}, $Na_2B_4O_7 \cdot 10H_2O$ 100 mg l^{-1}, $CuSO_4 \cdot 5H_2O$ 250 mg l^{-1} and $Na_2MoO_4 \cdot 2H_2O$ 50 mg l^{-1}.

Suppl. Table S3 Compositions of the cultivation media used in the PRSR and PRSN experiments (Boruta et al. 2023a, 2023b)

Medium component	Experiment								
	PRSR1	PRSR2	PRSR3	PRSN1	PRSN2	PRSN3			
Glucose (g l^{-1})	10	10	10	10	10	10			
Lactose (g l^{-1})	40	40	40	40	40	40			
Yeast extract (g l^{-1})	4	4	4	4	4	4			
$(NH_4)_2SO_4$ (g l^{-1})	0	2	2	0	0	0			
KH_2PO_4 (g l^{-1})	1.51	1.51	1.51	1.51	1.51	1.51			
NaCl (g l^{-1})	0.4	0.4	0.4	0.4	0.4	0.4			
$MgSO_4 \cdot 7H_2O$ (g l^{-1})	0.5	0.5	0.5	0.5	0.5	0.5			
Biotin (mg l^{-1})	0.04	0.04	0.04	0.04	0.04	0.04			
Phenylacetic acid (g l^{-1})	0.25	0.25	0.25	0.25	0.25	0.25			
Trace elements solution (ml l^{-1})*	1	1	1	1	1	1			

*Trace element solution of the following composition: $MnSO_4$ 50 mg l^{-1}, $ZnSO_4 \cdot 7H_2O$ 1 g l^{-1}, $Fe(NO_3)_3 \cdot 9H_2O$ 2 g l^{-1}, $Na_2B_4O_7 \cdot 10H_2O$ 100 mg l^{-1}, $CuSO_4 \cdot 5H_2O$ 250 mg l^{-1} and $Na_2MoO_4 \cdot 2H_2O$ 50 mg l^{-1}.

Suppl.Table S4 Inoculation method in runs ATSR, ASTN, PRSR, PRSN (Boruta et al. 2021, 2022, 2023a, 2023b)

**Exp-eri-mental run**	**Variant in the run**	**Type and volume of inoculum**	**Time of inoculation of species**
ATSR1<br>ATSR2<br>ATSR3<br>ATSR4<br>ATSR5<br>ATSR6	*A. terreus* monoculture (bioreactor #1)	*A. terreus* preculture (300 ml)	0 h
	S. rimosus monoculture (bioreactor #2)	*S. simosus* preculture (300 ml)	0 h
	A. terreus + *S. rimosus* coculture (bioreactor #3)	*S. rimosus* preculture (300 ml) + *A. terreus* spore suspension (300 ml)	0 h
ATSR7	*A. terreus* monoculture (bioreactor #1)	*A. terreus* preculture (300 ml)	0 h
	S. rimosus monoculture (bioreactor #2)	*S. simosus* preculture (300 ml)	24 h
	A. terreus + *S. rimosus* coculture (bioreactor #3)	*S. rimosus* preculture (300 ml) + *A. terreus* preculture (300 ml)	0 h *A. terreus* 24 h *S. rimosus*
ATSR8	*A. terreus* monoculture (bioreactor #1)	*A. terreus* preculture (300 ml)	0 h
	S. rimosus monoculture (bioreactor #2)	*S. simosus* preculture (300 ml)	24 h
	A. terreus + *S. rimosus* coculture (bioreactor #3)	*S. rimosus* preculture (300 ml) + *A. terreus* preculture (300 ml)	0 h *A. terreus* 24 h *S. rimosus*
ATSR9	*A. terreus* monoculture (bioreactor #1)	*A. terreus* spore suspension (300 ml)	0 h
	S. rimosus monoculture (bioreactor #2)	*S. simosus* spore suspension (300 ml)	0 h

	A. terreus + *S. rimosus* coculture (bioreactor #3)	*S. rimosus* spore suspension (300 ml) + *A. terreus* spore suspension (300 ml)	0 h
ATSN3	*A. terreus* monoculture (bioreactor #1)	*A. terreus* spore suspension (300 ml)	0 h
	S. noursei monoculture (bioreactor #2)	*S. noursei* spore suspension (300 ml)	0 h
	A. terreus + *S. noursei* coculture (bioreactor #3)	*S. noursei* spore suspension (300 ml) + *A. terreus* spore suspension (300 ml)	0 h
ATSN4	*A. terreus* monoculture (bioreactor #1)	*A. terreus* spore suspension (300 ml)	0 h
	S. noursei monoculture (bioreactor #2)	*S. noursei* spore suspension (300 ml)	0 h
	A. terreus + *S. noursei* coculture (bioreactor #3)	*S. noursei* spore suspension (300 ml) + *A. terreus* spore suspension (300 ml)	0 h
ATSN5	*A. terreus* monoculture (bioreactor #1)	*A. terreus* preculture (300 ml)	0 h
	S. noursei monoculture (bioreactor #2)	*S. noursei* preculture (300 ml)	0 h
	A. terreus + *S. noursei* coculture (bioreactor #3)	*S. noursei* preculture (300 ml) + *A. terreus* preculture (300 ml)	0 h
ATSN6	*A. terreus* monoculture (bioreactor #1)	*A. terreus* preculture (300 ml)	0 h
	S. noursei monoculture (bioreactor #2)	*S. noursei* preculture (300 ml)	0 h
	A. terreus + *S. noursei* coculture (bioreactor #3)	*S. noursei* preculture (300 ml) + *A. terreus* preculture (300 ml)	0 h
ATSN7	*A. terreus* monoculture (bioreactor #1)	*A. terreus* preculture (300 ml)	24 h
	S. noursei monoculture (bioreactor #2)	*S. noursei* preculture (300 ml)	0 h
	A. terreus + *S. noursei* coculture (bioreactor #3)	*S. noursei* preculture (300 ml) + *A. terreus* preculture (300 ml)	24 h *A. terreus* 0 h *S. noursei*
ATSN8	*A. terreus* monoculture (bioreactor #1)	*A. terreus* preculture (300 ml)	0 h

	S. noursei monoculture (bioreactor #2)	*S. noursei* preculture (300 ml)	24 h
	A. terreus + *S. noursei* coculture (bioreactor #3)	*S. noursei* preculture (300 ml) + *A. terreus* preculture (300 ml)	0 h *A. terreus* 24 h *S. noursei*
PRSR1	*P. rubens* monoculture (bioreactor #1)	*P. rubens* spore suspension (300 ml)	0 h
	S. rimosus monoculture (bioreactor #2)	*S. rimosus* spore suspension (300 ml)	0 h
	P. rubens + *S. rimosus* coculture (bioreactor #3)	*S. rimosus* spore suspension (300 ml) + *P. rubens* spore suspension (300 ml)	0 h
PRSR2	*P. rubens* monoculture (bioreactor #1)	*P. rubens* spore suspension (300 ml)	0 h
	S. rimosus monoculture (bioreactor #2)	*S. rimosus* spore suspension (300 ml)	24 h
	P. rubens + *S. rimosus* co-culture (bioreactor #3)	*S. rimosus* spore suspension (300 ml) + *P. rubens* spore suspension (300 ml)	0 h *P. rubens* 24 h *S. rimosus*
PRSR3	*P. rubens* monoculture (bioreactor #1)	*P. rubens* spore suspension (300 ml)	0 h
	S. rimosus monoculture (bioreactor #2)	*S. rimosus* spore suspension (300 ml)	48 h
	P. rubens + *S. simosus* coculture (bioreactor #3)	*S. rimosus* spore suspension (300 ml) + *P. rubens* spore suspension (300 ml)	0 h *P. rubens* 48 h *S. rimosus*
PRSN1	*P. rubens* monoculture (bioreactor #1)	*P. rubens* spore suspension (300 ml)	0 h
	S. noursei monoculture (bioreactor #2)	*S. noursei* spore suspension (300 ml)	0 h
	P. rubens + *S. noursei* coculture (bioreactor #3)	*S. noursei* spore suspension (300 ml) + *P. rubens* spore suspension (300 ml)	0 h
PRSN2	*P. rubens* monoculture (bioreactor #1)	*P. rubens* spore suspension (300 ml)	0 h
	S. noursei monoculture (bioreactor #2)	*S. noursei* spore suspension (300 ml)	24 h

	P. rubens + *S. noursei* coculture (bioreactor #3)	*S. noursei* spore suspension (300 ml) + *P. rubens* spore suspension (300 ml)	0 h *P. rubens*<br>24 h *S. noursei*
PRSN3	*P. rubens* monoculture (bioreactor #1)	*P. rubens* spore suspension (300 ml)	0 h
	S. noursei monoculture (bioreactor #2)	*S. noursei* spore suspension (300 ml)	48 h
	P. rubens + *S. noursei* coculture (bioreactor #3)	*S. noursei* spore suspension (300 ml) + *P. rubens* spore suspension (300 ml)	0 h *P. rubens*<br>48 h *S. noursei*

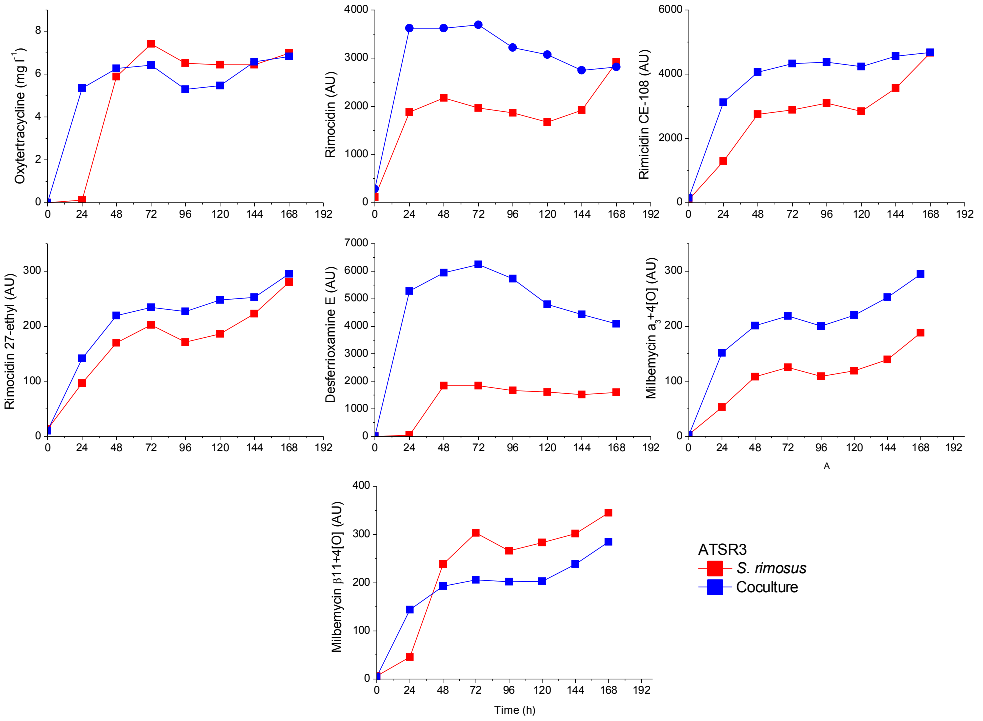

Suppl. Fig. S1 Metabolic dominance of *S. rimosus* shown on the example of nine secondary metabolites despite lack of its kinetic dominance in ATSR3; upon selected data from Boruta et al. (2021)

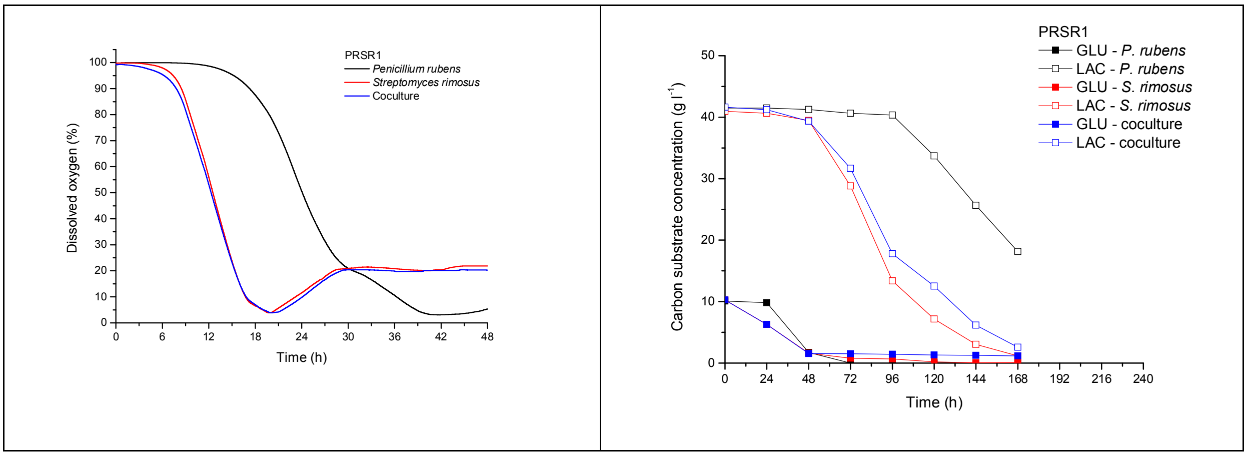

Suppl. Fig. S2 Kinetic dominance of *S. rimosus* over *P. rubens* in PRSR1; upon selected data from Boruta et al. (2023a)
